# Extracellular vesicles from *Echinococcus granulosus* larval stage: Isolation, characterization and uptake by dendritic cells

**DOI:** 10.1371/journal.pntd.0007032

**Published:** 2019-01-07

**Authors:** María Celeste Nicolao, Christian Rodriguez Rodrigues, Andrea C. Cumino

**Affiliations:** 1 Laboratorio de Zoonosis Parasitarias, Departamento de Biología, Facultad de Ciencias Exactas y Naturales, Universidad Nacional de Mar del Plata (UNMdP), Funes, Nivel Cero, Mar del Plata, Argentina; 2 Consejo Nacional de Investigaciones Científicas y Técnicas (CONICET), Argentina; 3 Departamento de Química, Facultad de Ciencias Exactas y Naturales, Universidad Nacional de Mar del Plata (UNMdP), Funes, Nivel 2, Mar del Plata, Argentina; IRNASA, CSIC, SPAIN

## Abstract

The secretion of extracellular vesicles (EVs) in helminth parasites is a constitutive mechanism that promotes survival by improving their colonization and adaptation in the host tissue. In the present study, we analyzed the production of EVs from supernatants of cultures of *Echinococcus granulosus* protoscoleces and metacestodes and their interaction with dendritic cells, which have the ability to efficiently uptake and process microbial antigens, activating T lymphocytes. To experimentally increase the release of EVs, we used loperamide, a calcium channel blocker that increases the cytosolic calcium level in protoscoleces and EV secretion. An exosome-like enriched EV fraction isolated from the parasite culture medium was characterized by dynamic light scattering, transmission electron microscopy, proteomic analysis and immunoblot. This allowed identifying many proteins including: small EV markers such as TSG101, SDCBP, ALIX, tetraspanins and 14-3-3 proteins; proteins involved in vesicle-related transport; orthologs of mammalian proteins involved in the immune response, such as basigin, Bp29 and maspardin; and parasite antigens such as antigen 5, P29 and endophilin-1, which are of special interest due to their role in the parasite-host relationship. Finally, studies on the EVs-host cell interaction demonstrated that *E*. *granulosus* exosome-like vesicles were internalized by murine dendritic cells, inducing their maturation with increase of CD86 and with a slight down-regulation in the expression of MHCII molecules. These data suggest that *E*. *granulosus* EVs could interfere with the antigen presentation pathway of murine dendritic cells inducing immunoregulation in the host. Further studies are needed to better understand the role of these vesicles in parasite survival and as diagnostic markers and new vaccines.

## Author summary

Human cystic echinococcosis, caused by chronic infection with the larval stage of *Echinococcus granulosus*, affects over 1 million people worldwide. This helminth parasite secretes numerous excretory/secretory products that are in contact with host tissues where it establishes hydatid cysts. In this study, we comprehensively characterized extracellular vesicles (EVs) from *E*. *granulosus* protoscoleces and metacestodes, and demonstrated for the first time that the exosome-like vesicles from helminths can interact with host dendritic cells and carry several immunoregulatory proteins. This study provides valuable data on cestode-host immune communication. Nevertheless, further research on EVs is needed to fully understand their role in the parasite-host interface and obtain new data concerning their function as therapeutic markers and diagnostic tools.

## Introduction

Human echinococcosis is a zoonotic cestode disease caused by the larval stages of *Echinococcus* (family Taeniidae). It is considered as a re-emerging and neglected disease that causes serious chronic lung and liver diseases. The two *Echinococcus* species of greater public health importance and economic concern worldwide are *Echinococcus multilocularis*, responsible for alveolar echinococcosis (which is restricted to the Northern hemisphere), and *Echinococcus granulosus* responsible for cystic echinococcosis (which is globally distributed) [1, 2].

The larval stage of these parasites develops as metacestodes (fluid-filled cysts) in the viscera (mainly in the liver) of mammalian intermediate hosts. Metacestodes are formed by a thin cellular layer (germinal layer) from which protoscoleces (larval form that can develop either in an adult worm in the final host or in a secondary hydatid cyst in an intermediate host) bud. The cells of the germinal layer secrete the laminated layer, an acellular and carbohydrate-rich layer that surrounds the metacestode [2, 3]. The laminated layer, only present in the genus *Echinococcus*, appears to be a key component of the host-parasite interface, being involved in the maintenance of the cyst physical integrity and in the interaction with the host immune system [2, 4]. This structure is composed of mucins containing defined galactose-rich carbohydrates, and, in *E*. *granulosus*, is also accompanied by calcium inositol hexakisphosphate deposits (InsP6) [3].

These helminth parasites lack digestive and excretory systems but have developed active endocytic and exocytic cellular processes to regulate metabolite uptake and excretion [5]. In previous studies, we have determined an increased exocytosis rate in the *E*. *granulosus* larval stage, which could be controlled by calcium concentration and in which proteins such as calcineurin and calpains are involved [6]. These proteins have been reported to be involved in unconventional vesicle-mediated protein secretion and in inflammatory responses [7]. Recently, it has been suggested that the endo/exosomal vesicular trafficking pathways share common features with autophagy [8, 9], which is an active process in *E*. *granulosus* both in basal conditions and after pharmacological treatment [10, 11].

Helminth parasites release several molecules, such as proteases, glycolytic enzymes and protease inhibitors into the mammalian hosts [12]. These products are known as excretory/secretory products which are exposed to the host immune system and could be involved in its modulation and the consequent parasite survival [13]. In this context, extracellular vesicles (EVs) are considered interesting target structures due to their potential role in parasite-parasite and host-parasite communication [14, 15]. Depending on their intracellular site of origin, composition and size, EVs are classified into exosomes, ectosomes or microvesicles, and apoptotic and autophagic vesicles [16–18]. Although EVs exhibit a varied range of sizes, exosomes are considered small vesicles (sEVs) of typically 30–150 nm which originate from the inward budding of late endosomes that form multivesicular bodies (MVBs) with intraluminal vesicles (ILVs) [19]. When MVBs fuse with the plasma membrane, ILVs are released as exosomes from the cell surface. Consequently, the biochemical composition of exosomes is associated with their biogenesis, including proteins from the endosomal-sorting complexes required for transport (ESCRT) pathway [20]. Although, the EV composition is presumably context-dependent, no universal and specific EV markers are yet available [21]. Nevertheless, Kowall et al. [22] have recently proposed the proteins Syntenin-1 (Syndecan Binding Protein -SDCBP-) and Tumor Susceptibility Gene 101 (TSG101) as markers of *bona fide* exosomes in mammalian systems.

Microvesicles comprise larger structures than exosomes (usually 100–1000 nm) and are directly produced by budding from the plasma membrane, generally as a consequence of an external stimulus that causes an intracellular Ca^+2^ increase [23].

Several studies have reported that helminth parasites secrete EVs that could play important roles during infection [24–28]. It is known that cestodes such as *Taenia crassiceps*, *Mesocestoides corti* and *E*. *multilocularis* secrete EVs with protein and miRNA cargo that can modulate the host immune system [29]. Recently, dos Santos et al. [30] confirmed the presence of EVs in hydatid fluid from fertile and infertile metacestodes of *E*. *granulosus*, whereas simultaneously, Siles-Lucas et al. [31] demonstrated that *E*. *granulosus* cysts secrete exosome-like vesicles into the hydatid fluid and that these vesicles contain proteins involved in cyst survival. Since information on the function of EVs in this cestode is still limited, the aims of this study were to characterize the EVs produced by the larval stage of *E*. *granulosus* and to investigate the interaction of EVs with host cells, to find out whether this interaction plays a role in host immunomodulation. In addition, given that the release of EVs can depend on different stimuli like calcium increase and therapeutic treatment, we analyzed the occurrence of exosome-like vesicles and the protein composition of EVs released from control parasites and parasites treated with loperamide, a calcium channel agonist with anti-echinococcal effect [32].

## Methods

### Ethics statement

The animal study was carried out in agreement with National Health Service and Food Quality (SENASA) guidelines, Argentina and with the 2011 revised form of The Guide for the Care and Use of Laboratory Animals published by the U.S. National Institutes of Health. The Animal Experimental Committee at the Faculty of Exact and Natural Sciences, Mar del Plata University approved the experimental protocols (permit number: 2555-08-16).

### Experimental animals

Specific-pathogen free female CF-1 mice (28–35 g) were provided by the SENASA. A minimum number of animals were used in each experiment. The animals (five mice per cage) were kept under controlled laboratory conditions (temperature ±20°C, 12 hour light/12 hour dark with lights off at 8.00 p.m.). They were maintained with water and food *ad libitum*, monitored daily and placed in a clean cage with fresh sawdust every 3 days. *E*. *granulosus* metacestodes were obtained from the peritoneal cavity of mice injected with 1500 protoscoleces in suspension. For each experiment, the infected mice were anesthetized with ketamine-xylazine (50 mg/kg/mouse-5 mg/kg/mouse) and sacrificed by cervical dislocation at 6–8 months post infection. All efforts were made to minimize suffering.

### *In vitro* culture of protoscoleces and metacestodes

*Echinococcus granulosus* protoscoleces were obtained from lung and liver of infected cattle presented for routine slaughter at the abattoir in the province of Buenos Aires, Argentina. The viscera were transported to the laboratory where the hydatid cysts were aseptically opened to remove the laminar and germinal membranes along with the hydatid fluid and the protoscoleces. Protoscoleces were exhaustively washed in Phosphate Buffered Saline (PBS) and maintained in sterile conditions until *in vitro* culture. Protoscolex *in vitro* culture (n = 3,000/9.5cm^2^), and viability assays were carried out as previously described [33]. Briefly, they were cultured in medium 199 (Gibco) supplemented with antibiotics (penicillin, streptomycin, and gentamicin 100 μg/ml) and glucose (4 mg/ml) in Leighton tubes at 37°C without changing the medium. Vitality was determined by methylene blue exclusion test. Otherwise, *E*. *granulosus* metacestodes (10–20 cysts for each drug treatment, with diameters ranging between 5 and 15 mm and free from the adventitial layer) were aseptically obtained from the peritoneal cavities of CF-1 mice 6–8 months after intraperitoneal infection with protoscoleces (n = 1500) [34]. They were cultured in the same conditions than protoscoleces and viability was assessed based on the collapse of the germinal layers.

Since it is known that intracellular calcium increase plays a role in exosome release [35–38], and the loperamide can rise the cytosolic free Ca^+2^ concentration ([Ca^+2^]_i_) [39], the addition of this drug at the parasite cultures, could ensure the high EVs production from *E*. *granulosus*. *In vitro* protoscolex- and metacestode-sub-lethal treatments were assayed with loperamide dissolved in dimethyl sulfoxide (DMSO) at 20 and 50 μM as final concentrations. Parasites incubated in culture medium containing 0.1% DMSO were used as controls.

### Detection of cytosolic calcium levels into *E*. *granulosus* protoscoleces

Changes in [Ca^+2^]_i_ using Fluo-3 acetoxymethyl ester (Fluo-AM) probe were fluorometrically monitored [33]. Experiments were performed with 5 x 10^3^ protoscoleces and incubated with 50 μM of loperamide for 4 h. Pretreatments with 1 mM EGTA plus 100 μM BAPTA-AM calcium chelators were performed for 15 min. Then, fluorescence was recorded with a spectrofluorimeter (model F-4500; Hitachi). The excitation and emission were set at 488 nm and 505–530 nm, respectively. Parasite-autofluorescence was individually corrected and untreated controls were included in each replication. Experiments were done in quintupled. Statistical analysis was done with the nonparametric Mann–Whitney test, a *p*-value of less than 0.05 was considered significant.

### Extracellular vesicles purification

Extracellular vesicles were enriched by differential centrifugation [40]. Briefly, 9000 protoscoleces or 45 cysts were maintained in serum-free media for 5 days and incubated in control conditions or with 20 μM of loperamide for 16 more hours. Following, the parasite culture medium was collected and centrifuged at 300 xg, 10 min; then at 2000 xg, 10 min and finally at 10000 xg for 30 min to remove large dead cells and large cell debris. The supernatant was ultracentrifuged at 100,000 xg for 1 h to pellet the vesicles in an Optima LE-80k ultracentrifuge (Beckman) using a 90 Ti rotor. To remove contaminating proteins, the pellet was washed with 3 ml of PBS and finally centrifuged at the same high speed. EVs were resuspended in 30 μl PBS and protein concentration was determined by absorbance at 280 nm with a Nanodrop spectrophotometer and by Bradford method for supernatants from the ultracentrifugation step. Statistical analysis was done with the Kruskal-Wallis test with Dunn's multiple comparisons post-test; *p*-values of < 0.05 were considered to be significant. Finally, EVs were stored at -80°C until experimental use. The 100,000 xg supernatant was collected, lyophilized, resuspended in 100 μl nuclease-free water and stored at -20°C for protein content comparison.

### Dynamic light scattering (DLS)

Size distribution profile and number of the vesicles isolated from protoscoleces of *E*. *granulosus* were performed with DLS using a Zetasizer Nano (Nano ZSizer-ZEN3600, Malvern, U.K.) at the Instituto de Investigaciones Fisicoquímicas Teóricas y Aplicadas (NIFTA- Argentina). Briefly, samples were captured at 25°C ± 1°C and diluted 1:50 in pre-filtered PBS and measure at a scattering angle of 173° with a He-Ne 633 nm laser. A total of 6 scans, each with duration of 60 s were recovered for each sample.

### Transmission electron microscopy

Extracellular vesicles were fixed in 2% paraformaldehyde in PBS and send refrigerated to a Transmission Electron Microscopy (TEM) external service for analysis (Centro Regional de Investigaciones Básicas y Aplicadas de Bahía Blanca -CRIBABB- Argentina). Procedures were carried out as described in [40]. EV-preparations were placed on a 300-mesh Formvar coated copper grids, negative stained with 1% (w/v) uranyl acetate for 1 min and examined at 100kV in a JEOL JSM 100CX II transmission electron microscope. Fiji software was used to evaluate the diameter of the vesicles observed in MET pictures. Data were statistically compared using the Kruskal-Wallis test with Dunn's multiple comparisons post-test; *p* values of < 0.05 were considered to be significant.

Additionally, control and loperamide-treated protoscoleces were also fixed and observed by TEM as previously described [11]. Briefly, parasites were fixed with 3% glutaraldehyde in sodium cacodylate buffer for 24 h at 4°C. Then, they were send refrigerated to a TEM external service for analysis (Servicio Central de Microscopía Electrónica de la Facultad de Ciencias Veterinarias, Universidad Nacional de La Plata) where they were post-fixed in 2% OsO_4_ in cacodylate buffer, dehydrated in a graded acetone series and subsequently embedded in resin epoxy and examined with a JEM 1200 EX II (JEOL Ltd., Tokio, Japan) transmission electron microscope at 80 kV.

### Proteomic analysis

The purified EVs (100 μg) and the supernatants were run 1 cm into the resolving gel of a 10% SDS-PAGE. Then, the gel was stained with colloidal Coomassie Blue G-250 and the samples cut from the gel and sent to the CEQUIBIEM proteomic service (Buenos Aires, Argentina) for mass spectrometry analysis and protein identification. Briefly, the samples were incubated with 20 mM dithiothreitol for 45 min at 56°C for reduction, and with 20 mM iodoacetamide for 45 min at room temperature in darkness for alkylation. Then, the samples were digested using trypsin and were processed by nano-HPLC (EASY-Spray Accucore, Thermo Scientific, West Palm Beach, FL, USA) coupled to a mass spectrometer with Orbitrap technology (Q-Exactive, Thermo Scientific, West Palm Beach, FL, USA) allowing peptide separation and identification. The sample ionization was made by electrospray (EASY-SPRAY, Thermo Scientific, West Palm Beach, FL, USA) and the data analysis was carried out by the Proteome Discoverer software version 1.4, Thermo Scientific. Only proteins with at least two peptides in two replicates were selected for further analyses.

The *in silico* analyses to establish the subcellular location and Gene Ontology (GO) classification of the identified proteins were performed using the UniProt database and software (http://www.uniprot.org/). Additionally, these proteins were also classified using the Reactome pathway database (https://reactome.org/) and manually based on data from the available literature. Moreover, the identified proteins were compared with those cataloged in the ExoCarta database (http://www.exocarta.org/).

The proteins identified as “uncharacterized, hypothetical, conserved or expressed protein” were analyzed and classified based on the presence of conserved domains using ProDom (http://prodom.prabi.fr/prodom/current/html/form.php), CDART (https://www.ncbi.nlm.nih.gov/Structure/lexington/lexington.cgi) and CDD (https://www.ncbi.nlm.nih.gov/cdd/). Finally, to determine whether the uncharacterized proteins were secreted by classical or nonclassical secretory pathways, we used SignalP server (http://www.cbs.dtu.dk/services/SignalP/) and SecretomeP server (http://www.cbs.dtu.dk/services/SecretomeP/) [41], respectively. These softwares were developed for bacterial and mammalian systems but they are also used in helminths [42, 43].

### Immunoblotting

Isolated EVs were lysed in CytoBuster protein extraction reagent (Novagen), supplemented with protease and phosphatase inhibitors (Thermo Fisher Scientific). Protein quantification was performed using the BCA Protein Assay (Pierce). A volume of 10 μl of EV-ultracentrifugated pellet (containing 30 μg of proteins in control samples and 69 μg of proteins in loperamide-treated samples) were loaded for all samples and analyzed simultaneously on 10% SDS-PAGE under non-reducing conditions. Polypeptides were electroblotted onto a nitrocellulose membrane (HyBond C; Amersham, Argentina) at 43 mA for 60 min. Following, the membranes were incubated in blocking solution (TBST: 20 mM Tris-HCl, 150 mM NaCl, 1% Tween-20, pH 7.6 containing 2% bovine serum albumin for 4 h at 20°C) and were probed with a 1:1000 dilution of mouse monoclonal antibodies raised against human CD9 (BD Pharmingen, clone M-L13) and CD63 (MEM-259, ImmunoTools clone MEM-259) to detect cross-reactivity with *E*. *granulosus* tetraspanins of approximately 25 and 50 kDa respectively. The corresponding antibodies datasheets do not allow the identification of the “antigen portion” used for their generation. Therefore, we analyzed the identity along the entire antigenic protein and the proteins of interest through sequence alignment. Also, we performed an antibody recognition ability analysis based on the identification of similar linear and conformational epitopes between human immunogen and parasite tetraspanins using BepiPred 2.0 (http://www.cbs.dtu.dk/services/BepiPred/) and CEP-Conformational Epitope Prediction Server- (http://196.1.114.49/cgi-bin/cep.pl). Finally, the blots were incubated with anti-rabbit immunoglobulin peroxidase-linked species-specific whole antibody (GE Healthcare, cat no. NA934V) and ECL reagents (GE Healthcare, cat no. RPN2106V1) to detect the chemiluminescent signal on film. A protein extract from human peripheral blood mononuclear cells (PBMCs) was used as a positive control.

### Extracellular vesicles membrane staining

Isolated EVs were labeled with PKH26 fluorescent dye (Sigma-Aldrich) according to the manufacturer’s instructions. In brief, 10 μl purified EVs were resuspended in 10 μl of Diluent C and mixed gently with PKH26 (added to a final concentration of 2 μM) in 150 μl of final volume for 35 min at 37° C in darkness. To stop the staining, they were incubated with BSA 1% for 10 min at room temperature. Then, the samples were washed with PBS followed by ultracentrifugation at 100,000 xg for 1 h to remove the excess dye and finally were resuspended in 150 μl of PBS. Negative controls consisted of the resuspended pellet after ultracentrifugation step labeled with the fluorescent dye alone, without purified EVs

### Generation and culture of Bone-Marrow dendritic cells

Bone Marrow-derived Dendritic Cells (BMDCs) were produced by flushing bone marrow of femurs and tibias of CF-1 mice (6–8 weeks old) as previously described with minor modifications [44]. Briefly, cells suspensions were depleted of erythrocytes with RBC lysing buffer (BD Bioscience, San Jose, CA). Cells were plated at 1 x 10^6^/ml in 6-well culture plates with 3 ml of supplemented RPMI 1640 (5% heat-inactivated fetal calf serum -Gibco; Invitrogen-, 100 U/ml penicillin/streptomycin, 10 μg/ml gentamicin and 2 mM L-glutamine, -all from Life Technologies, Grand Island, NY-). To induce DC-differentiation, cells were cultured in presence of 100 ng/ml Flt3-L (R&DSystems) at 37°C in 5% CO_2_ for 6 days. Finally, DC-population was characterized by flow cytometry using fluorescence-conjugated monoclonal antibodies (mAbs) directed against CD11b (M1/70), CD11c (HL3), CD3 (145-2C11), CD45R/B220 (RA3-6B2), SiglecH (eBio440c), CD172a (P84) and CD24 (M1/69) (eBiosciences, San Diego, CA). Approximately 70–90% of the cells were CD11c^+^.

### Analysis of the interaction between Bone-Marrow dendritic cells with extracellular vesicles from *E*. *granulosus*

Endocytosis of EVs by BMDCs and maturation assays was performed by flow cytometry and confocal microscopy. BMDCs (1 x 10^6^ cells/ml) were cultured with or without 150 μl PKH26 labeled-extracellular vesicles purified (10 μl pellets recovered by ultracentrifugation coming from culture supernatants of 3000 protoscoleces) from control or 20 μM loperamide-treated samples for 30 min at 37°C. Cells were then washed gently, pelleted and maintained for 18 h in culture before harvested. Incubation of BMDCs with EVs at 4°C was used as negative control of endocytosis. In addition, to determine DC maturation, the cells were stimulated for 18 h with 100 ng/mL of lipopolysaccharide (LPS, Sigma-Aldrich Co, positive control).

### Flow cytometry

Fluorescein isothiocyanate (FITC) or phycoerythrin-conjugated mAbs directed to CD11c (HL3), CD40 (HM40-3), CD80 (16-10A1), CD86 (GL1), MHC class I (AF6-88.5.5.3) and MHC class II (M5 / 114.15.2) were from eBioscience (San Diego, CA, USA). In all cases, isotype-matched control antibodies were used, and a gate (R1) was defined in the analysis to exclude all nonviable cells and debris, based on size and propidium iodide staining. The analysis was performed using a PartecCyflow Space (Sysmex, UK) flow cytometer, and the FlowJo software (Treestar). The results are expressed as the mean fluorescence intensity or as the percentage of positive cells. Data were statistically compared using the Kruskal-Wallis test with Dunn's multiple comparisons post-test; *p* values of < 0.05 were considered to be significant.

### Confocal microscopy

Immediately after incubation of the BMDCs with the EVs, cells were then harvested and plated on alcian blue-treated coverslips (12 mm) during 20 min at room temperature. Then, the cells were washed with PBS-BSA 2% in a wet chamber and fixed in 4% PFA and permeabilized with 0.05% saponin. Afterward, they were incubated with mAb MHC class II-FITC antibody (eBioscience, San Diego, CA, USA) for 1 h at 37°C, washed and incubated with 50 ng/ml DAPI (Sigma-Aldrich, USA) to counterstained nuclei. Coverslips were mounted on glass slides using Fluoromount G. Immunofluorescence and images were acquired with an inverted confocal laser scanning microscope (Nikon, Confocal Microscope C1) using a 60 x oil immersion objective with an excitation/emission wavelength 485/538 nm for FITC, 358⁄461 nm for DAPI and 551/567 nm for PKH26. Fluorescent intensity and co-localization analysis were performed using Histogram and Coloc 2 plugins in Fiji software. Briefly, to quantify MHCII modulation in cell surface, a total of ten cells in absence or presence of EVs was analyzed. Image files were loaded as separate image stacks. Then, surrounding background was subtracted before different region of interest (ROI) were analyzed to obtain the mean intensity values. For co-localization of MHCII molecules with EVs labeled with PKH26 the Pearson’s coefficient (r) was used to analyze the correlation of the intensity values of green and red pixels in dual-channel images. Statistical analysis was done with the nonparametric Mann–Whitney test, a *p*-value of less than 0.05 was considered significant.

## Results

### *Echinococcus granulosus* larval stage produces exosome-like vesicles which increase with [Ca^+2^]_i_ during loperamide treatment

**Fig 1:**
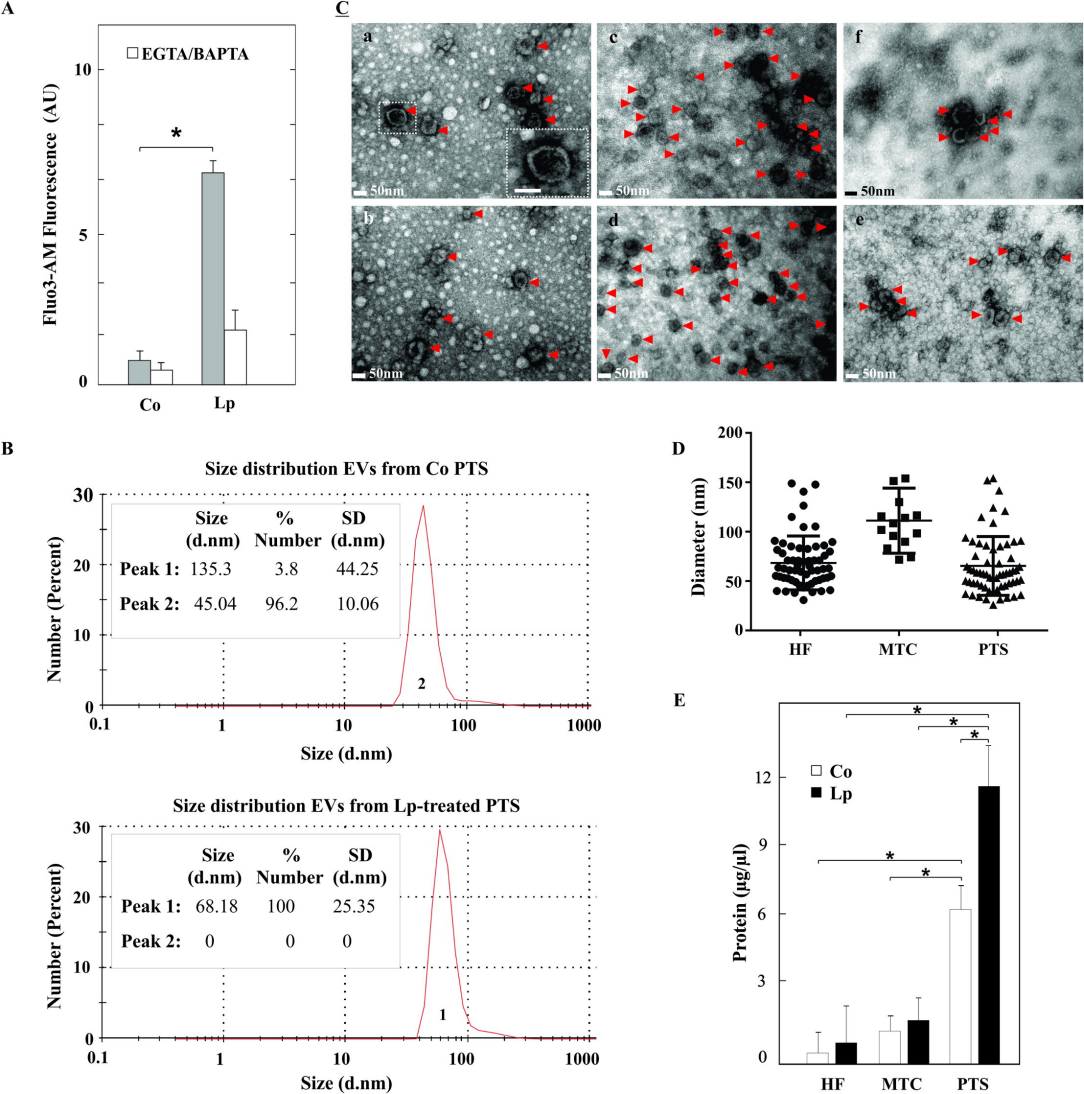
Characterization of extracellular vesicles purified from *E*. *granulosus* larval stage. **A.** Determination of changes in the cytosolic free Ca^+2^ concentration from control protoscoleces incubated with buffer (Co) or with 50 μM of loperamide (Lp) for 4 h and then loaded with Fluo-3-AM (grey bars). Pretreatments with 1 mM EGTA plus 100 μM BAPTA-AM for 15 min were carried out before the Fluo-3-AM incubations (white bars). Data are the mean ± S.D. from five independent experiments. Asterisk indicates significant differences (Mann–Whitney test, * p < 0.05). **B.** Size distribution curves determined by DLS of isolated EVs from control (Co) and loperamide (Lp)-treated protoscoleces (PTS). **C.** Morphological characterization of purified EVs through TEM. (a-b) Control PTS.(c-d) loperamide-treated PTS. (e) Control. MTC (f) Control hydatid fluid. Identification of abundant exosome-like structures indicated with arrowheads (size range from 25–150 nm in diameter and with the typical cup-shaped appearance-boxed area-). Scale indicates 50 nm. **D.** Estimation of EVs sizes from control protoscoleces (PTS), metacestodes (MTC) and hydatid fluid (HF) using Fiji software. **E**. Determination of protein concentration of ultracentrifugation pellet from seven independent assays. Data were presented as the mean ± SD. Asterisk indicates significant differences (Kruskal-Wallis with Dunn's post-test, * p < 0.05).

Since exocytosis could be regulated by [Ca^+2^]_i_, loperamide was used as an exocytic stimulus applying a concentration and time of incubation that guaranteed parasite viability [32]. Loperamide exposure (50 μM) increased free [Ca^+2^]_i_ 6-fold over a 4 h incubation period in comparison with the control (Fig 1A). The fluorescence signal diminished around control values after pretreatment with a mixture of EGTA (an extracellular chelator) plus BAPTA-AM (a membrane-permeable calcium chelator) in the medium.

In order to determine whether *E*. *granulosus* larval stage produces EVs, we followed a series of centrifugal steps of increasing speed as was described by Théry et al. [40] from which small EVs (sEVs or nanovesicles) were mainly isolated [22]. The identification and characterization of EVs focused on DLS for size distribution determination (Fig 1B) and TEM for morphology assessment (Fig 1C and 1D). The diameter of the majority of EVs was within the expected size range for exosome-like vesicles. The 96,2% of EVs from control protoscoleces was among 30–90 nm (45.04 ± 10.06 nm) and the 100% of EVs from loperamide-treated parasites showed a size range among 35–110 nm (68.18 ± 25.35 nm). However, a minor population of EVs from control protoscoleces (3.8%) shows a size of 135.3 ± 44.25 nm (Fig 1B). TEM analysis confirmed the presence of sEVs with the typical cup-shaped structures of 25–150 nm (Fig 1C and 1D) in accordance with DLS outcome. EVs from protoscoleces and hydatid fluid exhibit similar sizes while those from metacestodes display higher diameters even though they are not statistically significant (Fig 1D). Additionally, EVs were more abundant in protoscolex-cultures than in metacestode-cultures and their hydatid fluids indicated by protein concentration (6 ± 1 μg/μl, 1.1 ± 0.5 μg/μl and 0.7 ± 0.5 μg/μl, respectively, Fig 1C and 1E).

TEM pictures from entire protoscoleces also show structures compatible with EVs (exosome-like vesicles, microvesicles) and MVBs with ILVs associated with the tegument and surface of protoscoleces (S1 Fig). Besides, sub-lethal treatment of protoscoleces with the drug induced ultrastructural changes in the tegument, such as disorganization of the distal cytoplasm and lack of glycocalyx and microtriches, while it was unaltered in control condition (S1 Fig).

### Small extracellular vesicles of *E*. *granulosus* larval stage contain characteristic proteins of exosome-like vesicles

**Fig 2:**
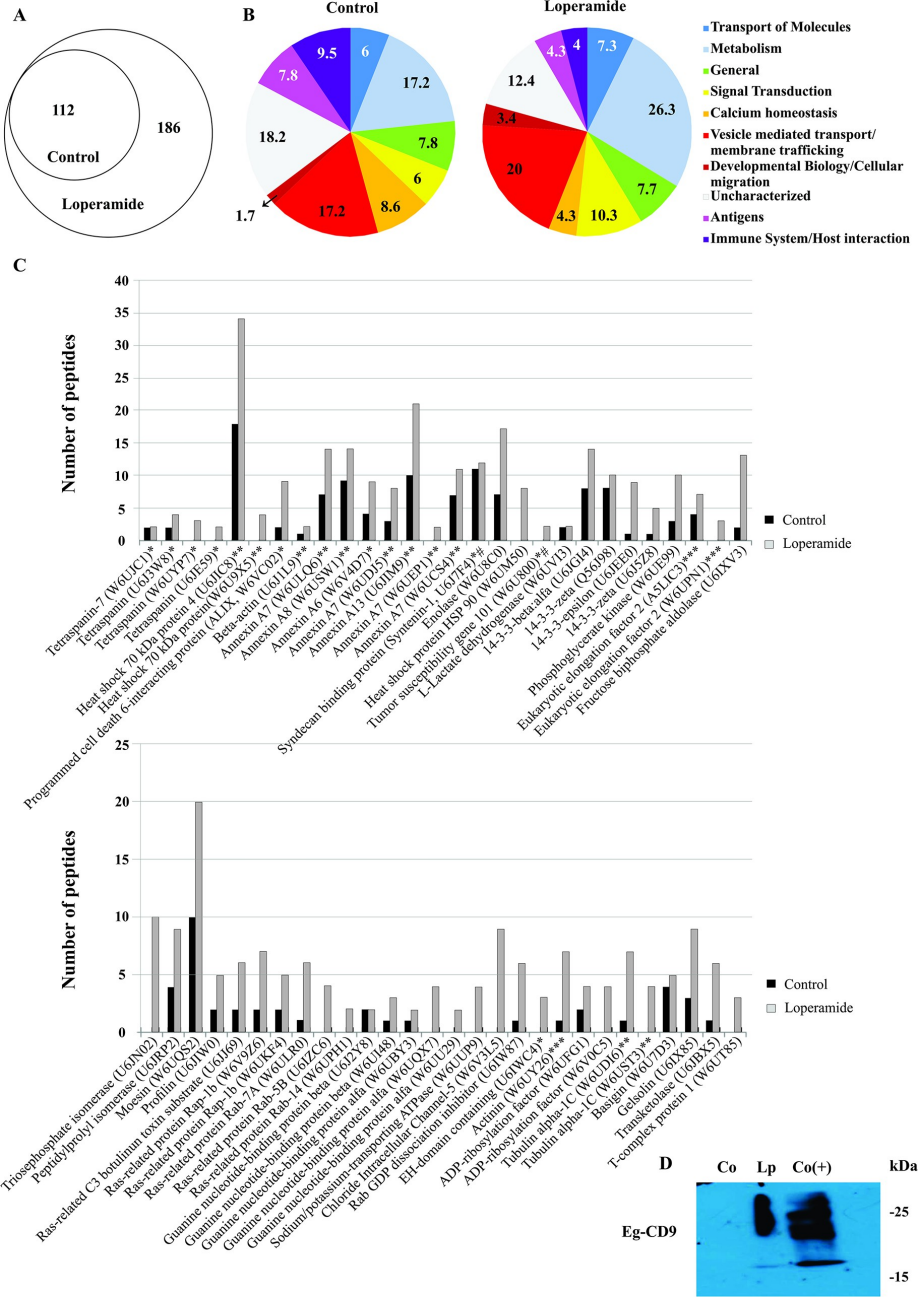
Proteomic analysis from extracellular vesicles of protoscoleces. **A.** Venn diagram of total proteins from control and loperamide-treated samples. **B.** Functional classification of proteins from control condition and proteins exclusively expressed under loperamide treatment. **C.** Proteins expressed in exosome-like vesicles from *E*. *granulosus* protoscoleces according to ExoCarta. #, exosomal markers; * small EVs specific proteins; **, proteins present in multiple EVs; ***, proteins present in large EVs. **D.** Western Blot of CD9 tetraspanin from control (Co) and loperamide (Lp)-treated samples revealed with a heterologous antibody against human CD9 (with expected size of approximately 25 kDa). Co (+): positive control (protein extract from PBMCs). Right lane show polypeptide sizes.

To characterize the protein-cargo and analyze the possible differences between the EVs obtained from untreated- and loperamide-treated parasites we carried out a proteomic analysis of the sEV-enriched fraction of parasites incubated in both conditions. In samples from metacestodes, we identified a very low number of proteins due to a low recovery of total proteins. Nevertheless, we could successfully identify 5 and 13 proteins in control and loperamide condition, respectively. Among these proteins, gelsolin, heat shock protein 70, tetraspanins and 14-3-3 protein which are usually present in exosomes, were identified. Otherwise, a total of 298 proteins were identified in samples of loperamide-treated protoscoleces. Of these proteins, 112 were common to both control and loperamide-treated samples whereas the remaining 186 proteins were exclusively found in the treated sample (Fig 2A and S1 and S2 Tables). Proteins were classified into 10 categories based on the analysis with Reactome database and information from the literature. In spite of the fact that loperamide increased the identified-protein number and the release of EVs including exosome-like vesicles, the abundance of proteins involved in calcium homeostasis, immune system/host interaction and antigens was greater in control conditions. On the other hand, proteins involved in metabolism, transport of molecules, signal transduction, vesicle-mediated transport/membrane trafficking and developmental biology/cellular migration were expressed in higher proportion in loperamide samples respect to the control (Fig 2B). A detailed examination at peptide level revealed that most of the common proteins showed a peptide abundance of 2-fold and 3-fold enriched in drug-treated samples compared with untreated-samples, except for some proteins such as multidrug resistance proteins, Ca-ATPase and Antigen 5, where the expression was increased between 5-fold and 9-fold in the sEVs released from loperamide-treated parasites (S1 Table and S1 and S2 Files).

In addition, we observed a high prevalence of uncharacterized proteins which are of special interest for their putative role in the parasite-host interaction. They were analyzed and classified based on the presence of conserved domains using ProDom, CDART and CDD which allowed the identification of 7 putative antigenic proteins, 2 tetraspanins, 2 thioredoxin-like proteins, 1 galectin/galactose-binding lectin, among others (S3 Table). Besides, we performed a sequence analysis using SignalP and SecretomeP which revealed that 17% and 23% of uncharacterized proteins were secreted by classical and nonclassical secretory pathways, respectively (S3 Table).

Moreover, we compared our data with those of ExoCarta database. We observed that 56 out of 298 proteins share homology with the proteins listed in ExoCarta comprising some exosomal markers such as syntenin 1 and TSG101; proteins present in specific sEVs such as tetraspanins, ALIX, annexin A6 and EH-domain containing protein; proteins present in multiple EVs such as heat shock 70 kDa proteins, annexins, beta-actin and tubulin alpha-1C; and proteins present in large EVs such as eukaryotic elongation factor 2 and actinin (Fig 2C). We also identified other proteins that account for the presence of ectosomes such as Rho family of small GTPases (including RhoA and Cdc42) and proteins implicated in MVB trafficking such Rab family of small GTPases and SNARE proteins (S1 and S2 Tables). On the other hand, certain proteins were exclusively detected in EVs under loperamide-treatment such as TSG101 (exosomal marker tumour susceptibility), EPS8-like protein (as part of epidermal growth factor receptor (EGFR) signalling), prominin-1 (CD133, a pentaspan protein) and the transforming growth factor-beta-induced protein ig-h3 (TGFBI, also known as keratoepithelin).

Finally, tetraspanins, which are very prevalent in exosomes and are involved in their biosynthesis were assayed by immunoblot analysis. The anti-CD9 and anti-CD63 antibodies used are directed against proteins which showed 26–30% or 21–23% amino acid identity with the four Eg-tetraspanins identify in our proteomic analysis (EUB61600 and EUB60810 in control and loperamide-samples and EUB63772 and EUB54099 only from loperamide-samples). Additionally, the antigenic regions of both human CD9 and the Eg-tetraspanins are coincidental, exposed, and with coil structure, suggesting that the probability of recognition by anti-CD9 include the four parasite proteins.

The transmembrane tetraspanins homolog to human CD9 (with an expected size of approximately 25 kDa) were enriched in the EV preparations from loperamide-treated samples (Fig 2D). The immunodetection of tetraspanins in drug-treated samples was surely due to higher protein loading of this sample in relation with the control. The sub-optimal protein concentration from control samples could result in the observed undetected signal.

### Extracellular vesicles of *E*. *granulosus* are internalized by dendritic cells and induce their maturation

**Fig 3:**
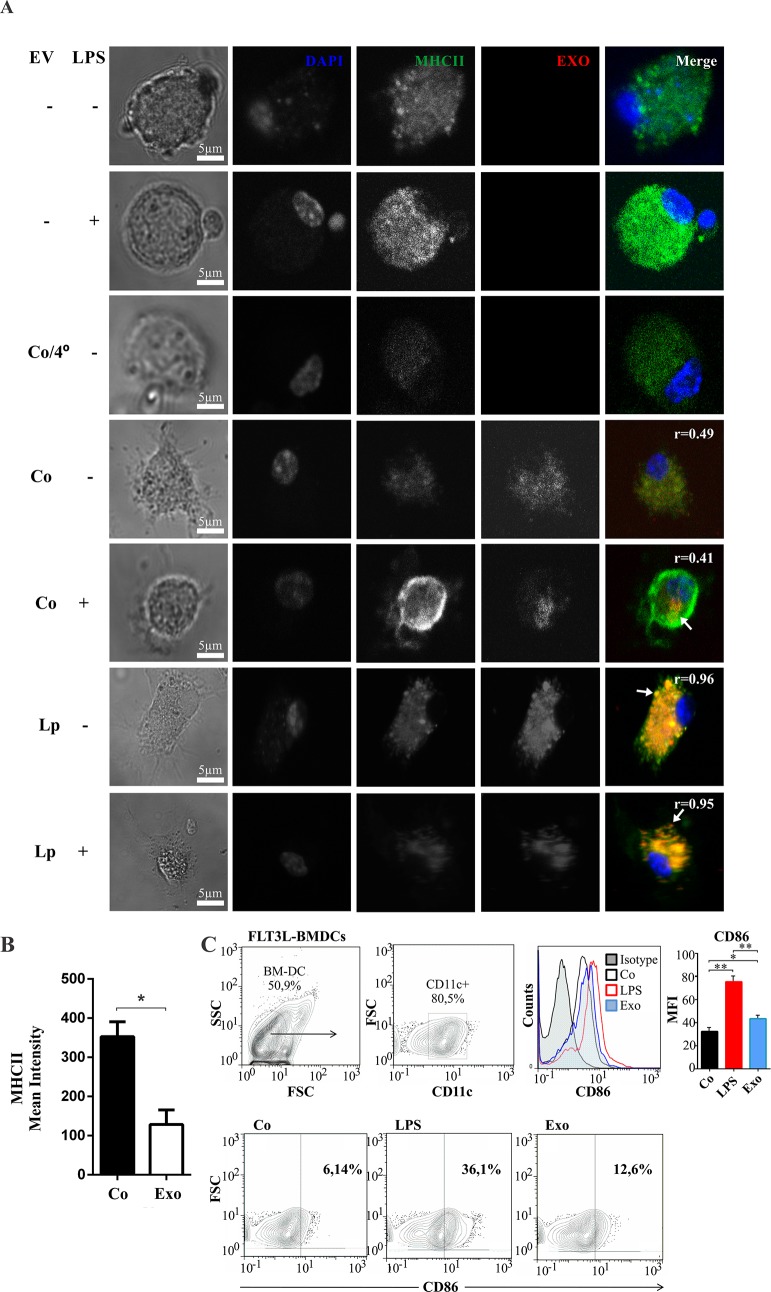
Determination of extracellular vesicles-host cell interaction. 1x10^6^ cells/ml Bone Marrow Dendritic Cells (BMDCs) were obtained after 6 days of culture in presence of FLT3-L. **A**. Immunofluorescence confocal microscopy (ICM) of BMDCs stimulated with LPS (100 ng/ml) or with 10 μl of EVs purified from control or 20 μM loperamide-treated protoscoleces of *E*. *granulosus* for 18 h to visualize MHCII-FITC (green), EVs-PKH26 (red), DAPI (blue). Arrows point the co-localization area. r = Pearson’s correlation coefficient for co-localization of MHCII-FITC (green) and EVs-PKH26 (red). Scale bars  =  5 μM. **B.** MHCII quantification. Bars show means ± S. D. of MHCII fluorescence quantification from five photographs using Fiji software. Asterisk indicates significant differences (Mann–Whitney test, * p < 0.05). **C.** Dot-plots illustrating the purity of differentiated BMDCs based on the expression of CD11c. After 18 h, BMDCs maturation was analyzed by flow cytometry by studying the percentage of CD86^+^ cells or the difference in mean fluorescent intensity (MFI) by histograms. A representative experiment (n  =  3) is shown. Data were presented as the mean ± SD. Asterisk indicates significant differences (Kruskal-Wallis with Dunn's post-test, * p < 0.05, ** p < 0.01).

Dendritic cells (DCs) are the unique professional antigen-presenting cell able to link the initiation of an antigen-specific response to the microbial mediators forming a decisive interface between innate and adaptive immunity. When they capture foreign antigens in the periphery undergo phenotypic, functional and migratory changes that allow them to present processed antigenic peptides to naïve T cells. In this way, DCs handle the adaptive immune system in a proinflammatory or tolerogenic profile [45]. In this context, we performed a functional analysis to disclose potential roles of *E*. *granulosus* EVs in host-parasite interactions. To monitor the internalization of EVs, we stained them with the red-fluorescent lipophilic compound PKH26. Coincubation of 1 x 10^6^ BMDCs for 30 min at 37°C with labeled and extensively washed EVs revealed that these cells internalized the vesicles as can be seen by confocal microscopy analysis in Fig 3A. As expected, when EVs and BMDCs were incubated at 4°C, no ingestion of vesicles was observed due to the loss of fluidity and endocytic capacity of the plasma membrane. When we compare control with LPS-treated BMDCs we observed similar amounts, but a different cellular distribution of the vesicles internalized. When EVs were exposed to control BMDCs, the dye PKH26 show a homogeneous fluorescent pattern with some punctate dot structures (Fig 3A, S2 Fig). Remarkably, the internalized vesicles by LPS-treated BMDCs show a dotted pattern at the perinuclear region. In addition, higher levels of the PKH26 dye have been observed in BMDCs incubated with purified EVs coming from loperamide-treated protoscoleces. It is also important to note the colocalization of the EVs and MHCII indicated by the high Pearson’s R value (0.95–0.96) specially in EVs from treated parasites (Fig 3A). This suggests that they are located at the same cellular site, probably related to endosomal-lysosomal compartments where preformed MHCII molecules are stored. Addiotionally, we performed a control to evaluate the labeling specificity using the dye purified without EVs which revealed only a 3–4% of cells with diffuse signal and nonspecific extracellular fluorescence in comparison with the 40% positive labeled cells observed in presence of EVs (S2 Fig). Due to vesicles secreted by *E*. *granulosus* also transport antigenic proteins, we, therefore, use the MHCII and the costimulatory molecule CD86 as markers of the maturation of DCs. A slight down-regulation in the expression of MHCII was evidenced by confocal microscopy when BMDCs were in contact with purified EVs from control protoscoleces (Fig 3A and 3B). Conversely, FACS analysis showed that the *E*. *granulosus* EVs induce the up-regulation of CD86 whereas similar expression was observed for CD40, CD80, MHCI and MHCII in a unified CD11c^+^ DCs population (Fig 3C and S3 Fig). In our assays, we have detected 3 different subpopulations of dendritic cells. Plasmacytoid dendritic cells (pDC, CD11c^+^ B220^+^ SinglecH^+^) and conventional dendritic cell (cDC): CD11b^+^ like DCs (CD11c^+^, CD172a^+^) and CD8^+^like DCs (CD11c^+^ CD24^+^ and CD172a^-^). However, the proportion of pDCs (with mainly antiviral and antitumor activity) was always less than 10%, and the 3 populations analyzed separately did not show any changes in the response against the stimulation of the exosomes-like from *E*. *granulosus*.

## Discussion

Extracellular vesicles have been widely related to the host-parasite relationship and cell comunication and are considered relevant for the infection and persistence of the parasite in the host [15, 46]. In this work, we isolated and characterized for the first time the EVs produced in *in vitro* cultures of *E*. *granulosus* protoscoleces and metacestodes obtained from infected mice, and we analyzed their biological function after contact with host DCs. In the course of the production of this manuscript, two studies evidenced EVs in hydatid fluid [30,31] and another one evidenced EV secretion by *E*. *multilocularis* [29].

As mentioned in the Results section, we isolated sEVs from the larval stage of *E*. *granulosus* which were similar in size, morphology (Fig 1) and protein content (Fig 2, S1 and S2 Tables) to the exosomes characterized in reports refered to *Echinococcus* sp., other parasites (*Echinostoma caproni*, *Heligosomoides polygyrus*, *Fasciola hepatica*, *Schistosoma japonicum*, *Taenia crassiceps*, *Mesocestoides corti*, among ohers) and mammals [24–26, 28–31, 47].

Dynamic light scattering assay and TEM images show exosome-like structures between 25 and 150 nm in diameter, which displayed a usual cup-shaped morphology (Fig 1B and 1C), thus allowing us to confirm the presence of exosome-like vesicles. Besides, mass spectrometry allowed us to identify a number of well-known exosomal and sEV markers, including TSG101, SDCBP and ALIX (Fig 2C, S1 and S2 Tables). Also, in accordance with previous reports, our results of immunoblot showed that these EVs were enriched in CD9-like tetraspanin, which could have a role in stabilizing membrane microdomains and increasing exosome production (Fig 2D) [22, 48]. Although some authors have previously identified CD63-like tetraspanins from *E*. *granulosus* [49, 50], in the present study we were not able to detect them under our western blot conditions probably due to a low antibody cross-reactivity against parasite tetraspanins. These proteins have been suggested as promising targets for vaccination or anti-parasitic therapy against *E*. *multilocularis* and *Opisthorchis viverrini*, respectively [51, 52]. In the present study, proteomic analysis allowed us to identify 112 and 298 proteins from EVs of control and loperamide-treated protoscoleces respectively of which 38 and 56 were specifically enriched in orthologs of mammalian proteins present in exosomes, respectively (Fig 2A and 2C). Additionally, they were compared with those previously reported for *Echinococcus* and other flatworm parasites (S1 and S2 Tables) revealing that the majority of components found in EVs (such as exosomal markers, proteins involved in MVB biogenesis, vesicle trafficking, enzymes, chaperones and cytoskeleton proteins) and several antigens were common among helminth parasites [26, 28, 29, 31]. Coincidentally with Siles Lucas et al. [31] we found in the ten most common proteins SDCBP, ezrin/moesin/radixin and antigen 5 (S1 and S2 Tables). The proteins coming from the membrane and cytosol included: heat shock proteins, signal or scaffolding proteins, endosome-membrane proteins (Annexins and Rabs), Heteromeric G proteins and Rab effectors (otoferlin and synaptotagmin-like protein), which are associated with exosome biogenesis (Fig 2C) [20]. Given that these proteins are representative of the ESCRT dependent pathway, we propose that this could be the main route involved in the exosome biogenesis in the *Echinococcus* larval stage, as previously described for adult *F*. *hepatica* [28]. In this line of evidence, the TEM images of *E*. *granulosus* protoscoleces displayed in S1 Fig demonstrated the potential occurrence of structures similar to MVBs in the distal cytoplasm of the larvae and the presence of EVs. All these findings support the assumption that the isolated nanovesicles in our preparations are exosome-like vesicles. However, although we had a population enriched in nanovesicles (with absence of nuclear and mitochondrial proteins and presence of known and uncharacterized proteins belonging to non-classical secretory pathways, Fig 1B and S1–S3 Tables), our purification protocol lacked a gradient separation step. Thus, we cannot discard the presence of contaminating soluble proteins and other types of EVs secreted by the parasites. Indeed, we identified proteins present in microvesicles (indicated with asterisks in Fig 2C) and soluble proteins previously identified as excretory/secretory products in this cestode [53–55]. Probably, the observed increases in intracellular calcium trigger a signal cascade that stimulates the release of microvesicles from the tegumental surface, also increasing the ratio of these vesicles in loperamide-treated samples respect to the controls [23]. Supporting this idea, in loperamide-treated samples, we observed differential expression of proteins involved in microvesicle biogenesis such as ADP-ribosylation factor 6, as reported in *F*. *hepatica* (S2 Table) [28].

To boost EV release and to analyze their protein cargo in relation with untreated conditions, we added loperamide into the parasite cultures. This drug increased [Ca^+2^]_i_ in both *E*. *granulosus* larval forms and in other cell systems (Fig 1A) [39], enhanced the EV production in the cestode (Fig 1), as previously reported in C_2_ C_12_ myoblast cells [56], and affected the protein composition of the released exosome-like vesicles (Fig 2A and 2C, and S2 Table). Although total protein content was higher in loperamide-treated samples than in controls, the proportion of parasite antigens and calcium homeostasis proteins (otoferlin, PDCD6, dysferlin, among others) was lower than in controls (Fig 2A and 2B, S1 and S2 Tables). Therefore, the molecular characteristics of the EVs released from protoscoleces under physiological conditions suggest that these vesicles could mediate biological aspects of the parasitic life cycle involved in parasite-parasite and/or parasite-host interactions. On the other hand, proteins exclusively detected in sEVs under loperamide-treatment included TSG101, which has been previously reported to increase after cancer chemotherapy [57], and several other proteins such as EPS8-like protein, prominin-1, TGFBI and CDC42-interacting protein, which could induce migration and cell proliferation between the host and the parasite [58–60]. Besides, the EVs purified from drug-treated protoscoleces were enriched in multidrug resistance proteins and glutathione S-transferases, which could represent a potential mechanism of the parasite to reduce the chemotherapeutic effectiveness of the drug, as previously reported in cancer cells [57, 61, 62]. The analysis of sEVs obtained under loperamide treatment was helpful to corroborate that these sEVs were similar in size and quality to control sEVs even in presence of initial ultrastructural alterations (S1 Fig) encouraging further analysis of sEVs characterization using other antiechinococcal drugs to determine the occurrence of antigenic and/or immunoregulatory proteins.

Similarly to that observed by TEM in protoscolex cultures, the supernatants of *E granulosus* metacestode cultures were enriched in exosome-like vesicles (Fig 1Ce and 1D). In support of this, nanovesicles crossing the laminated layer of *Echinococcus* metacestodes have been described through ultrastructural analysis of the tegument [63, 64]. In the present study, the metacestodes used to obtain EVs were collected from the inner part of the cystic masses that are surrounded by the adventitial layer. This strategy allowed the isolation of cysts free of the collagen capsule and host cells and consequently of EVs only of parasite origin. Recently, it has been described that the metacestode stages of *T*. *crassiceps* and *M*. *corti* secrete EVs in close contact with the host, but that in *E*. *multilocularis* EVs are retained by the laminated layer [29]. The laminated layer of *E*. *multilocularis* is much thinner than that of *E*. *granulosus;* therefore, in the latter, the EVs would be mostly retained. Interestingly, the laminated layer of *E*. *granulosus* possesses crystalline granules containing deposits of the calcium salt of InsP6 (Ca_5_H_2_L_16H_2_O, where L represents fully deprotonated InsP6), which are absent in *E*. *multilocularis* [3]. This molecule has been reported to bind to numerous proteins that regulate intracellular vesicle traffic [65, 66]. It is known that a true exchange of macromolecules across of the laminated layer occurs between the host and the parasite, with constant vesicular trafficking through the tegument [3, 67]. This movement may depend on signature organellar targeting motifs within the proteins and on their interactions with certain components of the laminated layer. High-affinity InsP6-binding proteins include components of *E*. *granulosus* exosome-like vesicles such as synaptogmins, ATP-dependent RNA helicases (DExD/H-box protein family), pleckstrin, ezrin/radixin/moiesin, gelsolin and galectin (S1–S3 Tables) [68]. Thus, it could be considered that extracellular InsP6 binds to proteins on *E*. *granulosus* exosome-like vesicles, acting as a “dynamic anchorage” that promotes the passage across the laminated layer and thus accounting for the EVs observed in the metacestode culture medium (Fig 1Ce).

It is widely known that exosome-like vesicles, particularly those derived from helminth parasites, mediate the immune modulation through their protein-, lipid- and RNAs-cargo [24, 69, 70]. Proteins contained in helminth exosome-like vesicles can modify host responses to favor parasite survival, proliferation and dissemination [24, 28]. Thus, in the present study, we first analyzed the interaction of exosome-like vesicles with BMDCs since the latter can adsorb exosomes and thus modify T cell responses or internalize these vesicles by endocytosis, present antigens and modify their own function conditioning T cell responses [71, 72]. Bone Marrow Dendritic Cells internalized exosome-like vesicles from *E*. *granulosus* thus promoting their maturation (Fig 3). An interesting finding was that the exposure of the DCs to EVs induced an unconventional activation profile, with increase in CD86, but with a slight decrease in MHC class II molecules (Fig 3). The MHCII increase was only statistically significant by confocal microscopy analysis probably due to its ability to detect both intracellular and cell surface molecules in contrast to FACS analysis where only surface molecules can be detected (S3 Fig). These maturation pattern on the course of differentiation of DCs have been previously reported during the exposure to hydatid fluid or purified antigen B [73]. This activation profile could be related to the modulation of the parasite to avoid antigenic presentation favoring the scape to both immunosurveillance and an effective immune response.

Based on mass spectrometry analysis and *in silico* functional categorization of *E*. *granulosus* EVs, in the present study and increasing the data previously reported in *Echinococcus*, we identified several proteins with immunomodulatory functions such as a B-Cell Receptor Associated Protein 29 (Bp29), which negatively modulates the membrane expression of MHCI in HeLa cells [74]. Another protein well represented in the EVs analyzed was basigin (also known as Extracellular Matrix Metalloproteinase Inducer–EMMPRIN- or cluster of differentiation 147 -CD147-), a member of the immunoglobulin superfamily which acts as the principal receptor that mediates chemotaxis by cyclophilins and regulates the responsiveness of lymphocytes by inhibition of T cell proliferation [75, 76]. An orthologous protein to CD147, associated with the negative regulation of T-reg cells, has also been previously identified in *F*. *hepatica* [77]. Besides, here we identified a maspardin ortholog (known as MAST or ACP33 protein), which is highly conserved in metazoans and is able to bind to CD4, acting as a negative regulator in T cell activation [78]. In the same line of evidence, we detected numerous antioxidant proteins that could neutralize the oxidative free radicals generated by host phagocytic cells such as thioredoxin peroxidase and glutathione S-transferase which have been previously described in excretory/secretory products from *E*. *granulosus* protoscoleces and hydatid fluid [53] and comprise a major detoxification system in parasites [79, 80]. Also, we identified annexins, which act as glucocorticoid-regulated proteins that promote the resolution of inflammation by limiting neutrophil recruitment and production of proinflammatory mediators and inducing macrophage reprogramming toward an alternative phenotype [81–83]. These effects could explain the type 2 immune response observed in patients with cystic echinococcosis [73]. Other proteins found in our purified EVs were peptidyl-prolyl cis-trans isomerases which show potential immunomodulatory activity, as the alteration in DC function by cytokine production, which leads to expansion of CD4^+^ Treg cells in *Schistosoma mansoni* [84]. Also, we found proteins that showed identity with parasite tegumental proteins which could inhibit polymorphonuclear cell chemotaxis and induce IL-4-T lymphocytes and non-complement fixing antibodies (IgG4) in patients with cystic echinococcosis [85]. In this way, due to their ability to suppress the innate and adaptive immune response, EVs could also be valuable tools to improve inflammation-associated disease [24]. On the other hand, and opposed to the immunoregulatory response, certain antigenic proteins described in protoscoleces and metacestodes were detected in *E*. *granulosus* EVs. EVs transfer antigens to DCs and T lymphocytes more efficiently than soluble peptides [70]. In our proteomics data, we found 14-3-3 proteins which have been reported to provide 97% protection against *E*. *multilocularis* challenge infection in rodent and the production of a specific humoral response in rhesus macaque models, respectively [86, 87], the endophilin B1, which is highly expressed in the tegument of *Taeniidae* metacestodes and responsible for a strong immune recognition in sera from patients with cystic and alveolar echinococcosis and chronic neurocysticercosis [88], antigen 5, an immuno-dominant serine-protease with heparan sulphate proteoglycan- and calcium-binding sites, and antigen P-29 immunologically related to antigen 5 used as a marker for post-surgery surveillance of cystic echinococcosis patients [89, 90]. Antigen 5 is highly immunogenic in human infections although its highly cross-reactive glycan moieties may involve a parasite evasion mechanism [91–95]. Particularly, this antigen showed greater abundance in the EVs obtained from loperamide-treated parasites than in the controls. This increase correlates with higher positivity rates to antigen 5 test detected in albendazole-treated patients in comparison with untreated patients [96], probably indicating that the antigen 5 is released not only as a soluble protein but also associated with EVs.

In summary, our study showed for the first time the secretion of EVs in *E*. *granulosus* protoscoleces and metacestodes obtained in infected mice. Our study also demonstrated conserved size, shape and content of particular excretory/secretory proteins of EVs, suggesting an important role of EVs in the maturation process of DCs which are essential for the coordination of specific immune responses. Nevertheless, additional work is necessary to shed light on the functionality of these DCs stimulated with EVs from *E*. *granulosus* in antigen presentation, cytokine release, and activation of T cell population. It will also be interesting to elucidate the components of EVs and establish whether they can be used as diagnostic markers for parasitic diseases, as new vaccines, and/or as treatment tools [97].

## Supporting information

S1 Table Proteomic analysis of extracellular vesicles from control protoscoleces of *Echinococcus granulosus*. (DOCX)

S2 Table Differencial proteomic analysis of extracellular vesicles from loperamide-treated protoscoleces of *Echinococcus granulosus*. (DOCX)

S3 Table Uncharacterized protein and expressed conserved protein identified from *Echinococcus granulosus* extracellular vesicles. (DOCX)

S1 File Complete proteomic data set from extracellular vesicles from control protoscoleces of *Echinococcus granulosus*. (XLSX)

S2 File Complete proteomic data set from extracellular vesicles from loperamide-treated protoscoleces of *Echinococcus granulosus*. (XLSX)

S1 Fig Identification of vesicles in the tegument surface of protoscoleces by transmition electron microscopy. (a-b) Control (c-d) loperamide-treated protoscoleces (20 μM, 18 h). MVB, multivesicular bodies; ILV, intraluminal vesicles; EV and arrowhead, extracellular vesicle; n, nucleus; dc, distal cytoplasm; gl, glycocalyx; mi, microtriches.
(TIF)

S2 Fig Specificity of PKH26 staining to monitor extracellular vesicle uptake by dendritic cells. 1x10^6^ cells/ml Bone Marrow Dendritic Cells (BMDCs) obtained after 6 days of culture in presence of FLT3-L were incubated with EVs labeled with PKH26 (EVs +) or with PKH26 dye alone purified without EVs (EVs -). Almost 40% of cells incubated with labeled EVs show positive staining (homogeneous fluorescent pattern with punctate dot structures, indicated by arrowheads), while only 3% of them were stained with a diffuse pattern in presence of the dye alone (indicated by an arrowhead and a question mark). Boxed areas correspond to the amplified images.
(TIF)

S3 Fig Maturation profile of dendritic cells stimulated with extracellular vesicles. 1x10^6^ cells/ml Bone Marrow Dendritic Cells (BMDCs) were obtained after 6 days of culture in presence of FLT3-L. After 18 h, BMDCs maturation was analyzed by flow cytometry by studying the difference in mean fluorescent intensity (MFI) of CD40, CD80, MHCI and MHCII in the gate of CD11c+ cells by histograms. A representative experiment (n  =  3) is shown. Data were presented as the mean ± SD. No significant differences were detected using a Kruskal-Wallis with Dunn's post-test, * p < 0.05.
(TIF)
