# Supplementary material for: Extracellular vesicles from Echinococcus granulosus larval stage: Isolation, characterization and uptake by dendritic cells
Source: PLoS Negl Trop Dis. 2019 Jan 7;13(1):e0007032. doi: 10.1371/journal.pntd.0007032 (PMC6344059; doi:10.1371/journal.pntd.0007032)
Supplement: S1 Table — (DOCX) [file pntd.0007032.s001.docx]

**Supplementary Table S1.** Proteomic analysis of extracellular vesicles from control protoescoleces of *Echinococcus granulosus*.

| **Identified protein** | **Uniprot ID** | **GeneBank ID** | **Peptides** | **Gene ontology (GO)** | **Homologous detected in other cestodes** |
| --- | --- | --- | --- | --- | --- |
| Calpain A | U6J063 | EUB64462 | 25 | GO:0004198 calcium-dependent cysteine-type peptidase activity; GO:0005622 intracellular | Em, Mc, Tc (Ancarola et al., 2017) |
| Heat shock 70 kDa protein 4 | U6JIC8 | EUB56318 | 18 | GO:0005524 ATP binding | Em, Mc, Tc (Ancarola et al., 2017)  Eg (Siles-Lucas et al., 2017)  Fh (Cwikliski et al., 2015)  Ec (Marcilla et al., 2012) |
| Uncharacterized protein | W6U780 | EUB56231 | 13 | GO:0016021 integral component of membrane |  |
| Ubiquitin | W6USS6 | EUB64675 | 3 | GO:0044260 cellular macromolecule metabolic process; GO:0044424 intracellular part |  |
| Uncharacterized protein | W6UFB0 | EUB59571 | 12 | GO:0016021 integral component of membrane |  |
| Actin cytoplasmic A3 | U6JLF5 | EUB56079 | 12 | GO:0005524 ATP binding | Eg (Siles-Lucas et al., 2017)  Fh (Cwikliski et al., 2015)  Ec (Marcilla et al., 2012) |
| Uncharacterized protein | W6ULE5 | EUB61903 | 13 |  |  |
| Syndecan binding protein syntenin (SDCBP) | U6J7F4 | EUB62730 | 11 |  |  |
| Annexin | U6JIM9 | EUB64724 | 10 | GO:0005544 calcium-dependent phospholipid binding | Eg, Em, Mc, Tc (Ancarola et al., 2017) |
| Moesin/ezrin/radixin | W6UQS2 | EUB64035 | 10 | GO:0003779 actin binding; GO:0005856 cytoskeleton | Mc, Tc, Ts, (Ancarola et al., 2017) |
| Uncharacterized protein | W6U6P0 | EUB56903 | 5 |  |  |
| Phosphoenolpyruvate carboxykinase [GTP] | W6UHV2 | EUB57677 | 12 | GO:0004613 phosphoenolpyruvate carboxykinase (GTP) activity; GO:0008152 metabolic process | Em, Mc, Tc (Ancarola et al., 2017)  Eg (Siles-Lucas et al., 2017)  Fh (Cwikliski et al., 2015)  Ec (Marcilla et al., 2012) |
| Uncharacterized protein | W6U1N0 | EUB54938 | 5 | GO:0016021 integral component of membrane |  |
| Uncharacterized protein | W6U118 | EUB54805 | 6 |  |  |
| Actin, cytoplasmic 2 | W6UFA4 | EUB56832 | 10 | GO:0005524 ATP binding | Eg, Tc (Ancarola et al., 2017) |
| Annexin | W6ULQ6 | EUB59082 | 7 | GO:0005544 calcium-dependent phospholipid binding | Eg, Em, Mc, Tc (Ancarola et al., 2017) |
| Estrogen regulated protein EP45 | U6IXU7 | EUB63680 | 10 | GO:0005615 extracellular space |  |
| Annexin | W6USW1 | EUB64725 | 9 | GO:0005544 calcium-dependent phospholipid binding | Eg, Em, Mc, Tc (Ancarola et al., 2017) |
| 14-3-3 protein beta:alpha | U6JGI4 | EUB54777 | 8 | GO:0019904 protein domain specific binding | Eg (Siles-Lucas et al., 2017)  Fh (Cwikliski et al., 2015)  Ec (Marcilla et al., 2012) |
| Leucine-rich repeat-containing protein | W6UG49 | EUB60460.1 | 8 |  |  |
| Hydatid disease diagnostic antigen P-29 | W6V2P2 | AF078931_1 | 6 |  |  |
| 14-3-3 protein | Q56J98 | EUB62107 | 8 | GO:0019904 protein domain specific binding | Eg (Siles-Lucas et al., 2017)  Fh (Cwikliski et al., 2015)  Ec (Marcilla et al., 2012) |
| Thioredoxin peroxidase | U6IUN6 | EUB64940 | 5 | GO:0016209 antioxidant activity; GO:0045454 cell redox homeostasis | Eg (Siles-Lucas et al., 2017)  Fh (Cwikliski et al., 2015)  Ec (Marcilla et al., 2012) |
| Enolase | W6U8C0 | EUB57443 | 7 | GO:0004634 phosphopyruvate hydratase activity; GO:0008152 metabolic process; GO:0044424 intracellular part | Mc, Tc, Ts (Ancarola et al., 2017)  Eg (Siles-Lucas et al., 2017)  Fh (Cwikliski et al., 2015)  Ec (Marcilla et al., 2012) |
| Uncharacterized protein | W6VBX7 | EUB64349 | 6 | GO:0007017 microtubule-based process |  |
| Annexin A7 | W6UCS4 | EUB59085 | 7 | GO:0005544 calcium-dependent phospholipid binding |  |
| Ag5 | I1WXU1 | EUB60304  AFI71096 | 6 | GO:0004252 serine-type endopeptidase activity |  |
| Uncharacterized protein | W6UR49 | EUB64195 | 5 |  |  |
| Sodium/glucose cotransporter | W6UGH3 | EUB60605 | 4 | GO:0055085 transmembrane transport |  |
| Signal recognition particle protein | W6UF17 | EUB59451 | 6 | GO:0003924 GTPase activity; GO:0048500 signal recognition particle; GO:0006614 SRP-dependent cotranslational protein targeting to membrane |  |
| Ras-related protein RabJ | W6URG5 | EUB64330 | 4 | GO:0003924 GTPase activity |  |
| Ras-related protein O-RAL | W6V1I3 | EUB64797 | 5 | GO:0003924 GTPase activity; GO:0007165 signal transduction | Eg, Mc, Tc (Ancarola et al., 2017) |
| Peptidyl-prolyl cis-trans isomerase | U6JRP2 | EUB61757 | 4 | GO:0003755 peptidyl-prolyl cis-trans isomerase activity |  |
| Transforming protein RhoA | W6UDK8 | EUB59108 | 4 | GO:0003924 GTPase activity; GO:0007264 small GTPase mediated signal transduction | Eg, Mc, Tc (Ancarola et al., 2017) |
| BRO1 domain-containing protein BROX | W6US60 | EUB61207 | 5 |  | Em, Mc, Tc (Ancarola et al., 2017) |
| Cdc42 | U6IVD5 | EUB64783 | 3 | GO:0003924 GTPase activity; GO:0007264 small GTPase mediated signal transduction | Em, Tc (Ancarola et al., 2017)  Eg (Siles-Lucas et al., 2017)  Fh (Cwikliski et al., 2015) |
| Major vault protein | W6UEU0 | EUB56627 | 3 | GO:0030529 intracellular ribonucleoprotein complex; GO:0005737 cytoplasm | Eg, Em, Tc (Ancarola et al., 2017) |
| Transporter | W6UNL2 | EUB63240 | 5 | GO:0008324 cation transmembrane transporter activity; GO:0016021 integral component of membrane |  |
| Glyceraldehyde-3-phosphate dehydrogenase | W6V1T8 | EUB59849 | 5 | GO:0004365 glyceraldehyde-3-phosphate dehydrogenase (NAD+) (phosphorylating) activity; GO:0006096 glycolytic process | Eg, Mc, Tc, Ts, (Ancarola et al., 2017)  Eg (Siles-Lucas et al., 2017)  Fh (Cwikliski et al., 2015)  Ec (Marcilla et al., 2012) |
| Rab | U6JDZ7 | EUB54636 | 5 | GO:0003924 GTPase activity | Em, Mc, Tc (Ancarola et al, 2017)  Eg (Siles-Lucas et al., 2017)  Fh (Cwikliski et al., 2015) |
| Uncharacterized protein | W6UE44 | EUB59131 | 4 |  |  |
| Syntaxin-binding protein | W6U9S4 | EUB58123 | 5 | GO:0006904 vesicle docking involved in exocytosis | Eg, Tc (Ancarola et al., 2017) |
| Basigin | W6U7D3 | EUB57090 | 4 | GO:0016021 integral component of membrane |  |
| Uncharacterized protein | W6UJ45 | EUB58177 | 2 |  |  |
| Annexin | W6V4D7 | EUB60984 | 4 | GO:0005544 calcium-dependent phospholipid binding; GO:0016817 hydrolase activity, acting on acid anhydrides | Eg, Em, Mc, Tc (Ancarola et al., 2017) |
| IST1 protein | W6U2U0 | EUB55420 | 4 | GO:0006810 transport | Eg, Tc (Ancarola et al., 2017) |
| Multidrug resistance protein | W6UVB8 | EUB64561 | 4 | GO:0042626 ATPase activity, coupled to transmembrane movement of substances |  |
| Endophilin-A1 | W6UGD8 | EUB60036 | 3 | GO:0005737 cytoplasm |  |
| Chloride intracellular channel protein 5 | W6V3L5 | EUB60614 | 4 |  |  |
| Gelsolin | U6IX85 | EUB57056 | 3 | GO:0051015 actin filament binding |  |
| Antigen EG13 | W6UE73 | EUB59176 | 4 |  |  |
| Synaptotagmin-14 | W6VAV1 | EUB63909 | 4 |  |  |
| B-cell receptor-associated protein | W6U5T3 | EUB56465 | 3 | GO:0006886 intracellular protein transport; GO:0016021 integral component of membrane |  |
| Elongation factor 1-alpha | A5LIC3 | EUB62510 | 4 | GO:0003924 GTPase activity; GO:0005737 cytoplasm | Eg, Mc, Tc (Ancarola et al., 2017) |
| Peptidase inhibitor | W6UHA5 | EUB60436 | 2 | GO:0005576 extracellular region |  |
| Glutathione peroxidase | W6UCC3 | EUB58341 | 4 | GO:0004602 glutathione peroxidase activity |  |
| Uncharacterized protein | W6UCY9 | EUB59175 | 4 |  |  |
| Uncharacterized protein | W6U6B2 | EUB53882 | 2 |  |  |
| Uncharacterized protein | W6UX03 | EUB58029 | 3 | GO:0016021 integral component of membrane |  |
| Severin | W6UUI4 | EUB57054 | 4 | GO:0051015 actin filament binding | Eg (Siles-Lucas et al., 2017)  Fh (Cwikliski et al., 2015)  Ec (Marcilla et al., 2012) |
| Calcium-transporting ATPase | W6ULV1 | EUB62111 | 3 | GO:0005388 calcium-transporting ATPase activity; GO:0016021 integral component of membrane |  |
| Ubiquitin C | W6U595 | EUB56300 | 3 |  |  |
| Uncharacterized protein | W6UFB6 | EUB59798 | 2 | GO:0016021 integral component of membrane |  |
| Tyrosine-protein kinase | W6UPI5 | EUB63168 | 3 | GO:0004715 non-membrane spanning protein tyrosine kinase activity |  |
| Calcium-binding protein p22 | W6V843 | EUB62659 | 3 | GO:0005509 calcium ion binding | Em, Mc (Ancarola et al., 2017) |
| Tegumental protein | W6U648 | EUB56610 | 2 | GO:0030286 dynein complex; GO:0007017 microtubule-based process |  |
| Major egg antigen | W6UXT1 | EUB63392 | 2 |  |  |
| Putative vesicle-associated membrane protein | W6VBD9 | EUB64129 | 3 | GO:0016192 vesicle-mediated transport; GO:0016021 integral component of membrane |  |
| Phosphoglycerate kinase | W6UE99 | EUB59398 | 3 | GO:0004618 phosphoglycerate kinase activity; GO:0006096 glycolytic process | Eg, Mc, Tc (Ancarola et al., 2017)  Eg (Siles-Lucas et al., 2017)  Ec (Marcilla et al., 2012) |
| Annexin | W6UDJ5 | EUB59083 | 3 | GO:0005544 calcium-dependent phospholipid binding | Eg, Em, Mc, Tc (Ancarola et al., 2017) |
| Ras-related protein Rab-8B | W6USR3 | EUB64338 | 3 | GO:0003924 GTPase activity | Em, Tc (Ancarola et al., 2017  Eg (Siles-Lucas et al., 2017)  Fh (Cwikliski et al., 2015) |
| Tetraspanin-7 | W6UJC1 | EUB61600 | 2 | GO:0016021 integral component of membrane |  |
| Myoferlin | W6UYH7 | EUB63692 | 3 | GO:0016021 integral component of membrane | Eg, Hm, Mc, Tc (Ancarola et al., 2017)  Eg (Siles-Lucas et al., 2017)  Fh (Cwikliski et al., 2015) |
| Major egg antigen p40 | U6JBW8 | EUB62057 | 3 |  |  |
| Uncharacterized protein | W6U828 | EUB57310 | 3 | GO:0045454 cell redox homeostasis |  |
| Malate dehydrogenase, cytoplasmic | W6U831 | EUB56506 | 3 | GO:0030060 L-malate dehydrogenase activity | Mc, Tc, Ts (Ancarola et al., 2017)  Eg (Siles-Lucas et al., 2017)  Fh (Cwikliski et al., 2015)  Ec (Marcilla et al., 2012) |
| Endonuclease/exonuclease/phosphatase family domain-containing protein | W6U976 | EUB57560 | 2 | GO:0004519 endonuclease activity; GO:0004527 exonuclease activity |  |
| Uncharacterized protein | W6TZB6 | EUB54145 | 2 |  |  |
| Calpain | W6UGM0 | EUB60655 | 2 | GO:0004198 calcium-dependent cysteine-type endopeptidase activity; GO:0005622 intracellular | Em, Tc (Ancarola et al., 2017) |
| Integral membrane protein | U6J887 | EUB60085 | 2 | GO:0016021 integral component of membrane |  |
| Rho GDP-dissociation inhibitor | W6V5R2 | EUB61684 | 2 | GO:0005094 Rho GDP-dissociation inhibitor activity; GO:0005737 cytoplasm |  |
| Profilin | U6JIW0 | EUB61070 | 2 | GO:0003779 actin binding |  |
| Tetraspanin | U6J3W8 | EUB60810 | 2 | GO:0016021 integral component of membrane |  |
| Tegumental protein | U6JDG7 | EUB59351 | 2 | GO:0005509 calcium ion binding |  |
| Guanine nucleotide binding protein subunit beta | U6J2Y8 | EUB61828 | 2 | GO:0007165 signal transduction | Em, Tc (Ancarola et al., 2017) |
| Dynein light chain 1 cytoplasmic | W6UUJ4 | EUB64351 | 2 | GO:0030286 dynein complex; GO:0007017 microtubule-based process; GO:0005509 calcium ion binding | Eg, Em, Mc, Tc (Ancarola et al., 2017) |
| T-cell immunomodulatory protein | W6V8B8 | EUB62759 | 2 | GO:0016021 integral component of membrane |  |
| Tubulin beta-2 chain | W6V3X7 | EUB60769 | 2 | GO:0005200 structural constituent of cytoskeleton; GO:0007017 microtubule-based process ; GO:0003924 GTPase activity | Eg, Mc, Tc (Ancarola et al, 2017)  Eg (Siles-Lucas et al., 2017)  Fh (Cwikliski et al., 2015)  Ec (Marcilla et al., 2012) |
| Phospholipid-transporting ATPase | W6U370 | EUB54981 | 2 | GO:0004012 phospholipid-translocating ATPase activity; GO:0005802 trans-Golgi network | Eg (Siles-Lucas et al., 2017)  Fh (Cwikliski et al., 2015) |
| Protein DJ 1 | U6JIQ5 | EUB57723 | 2 |  |  |
| Alkaline phosphatase | W6U6M0 | EUB56860 | 2 | GO:0004035 alkaline phosphatase activity |  |
| F actin capping protein subunit beta | U6J0Y2 | EUB65105 | 2 | GO:0008290 F-actin capping protein complex; GO:0008064 regulation of actin polymerization or depolymerization |  |
| Ras-related protein Rap-1b | W6V9Z6 | EUB63534 | 2 | GO:0003924 GTPase activity; GO:0007165 signal transduction | Eg, Em, Mc, Tc (Ancarola et al., 2017) |
| Ras-related protein Rap-1b | W6UKF4 | EUB62005 | 2 | GO:0003924 GTPase activity; GO:0007165 signal transduction; GO:0016020 membrane | Eg, Em, Mc, Tc (Ancarola et al., 2017) |
| Anoctamin | U6JHY6 | EUB54990 | 2 | GO:0046983 protein dimerization activity; GO:0016021 integral component of membrane | Eg, Tc (Ancarola et al., 2017)  Eg (Siles-Lucas et al., 2017)  Fh (Cwikliski et al., 2015) |
| Beta-soluble NSF attachment protein | W6VCB5 | EUB64499 | 2 | GO:0006886 intracellular protein transport |  |
| Uncharacterized protein | W6U7G9 | EUB56306 | 2 |  |  |
| Syntaxin | U6J034 | EUB61288 | 2 | GO:0000149 SNARE binding; GO:0005484 SNAP receptor activity; GO:0017157 regulation of exocytosis; GO:0016021 integral component of membrane | Em, Tc (Ancarola et al., 2017) |
| Multidrug resistance protein | W6V0Y0 | EUB64562 | 2 | GO:0042626 ATPase activity, coupled to transmembrane movement of substances |  |
| Ras C3 botulinum toxin substrate 2 | U6JJ69 | EUB57999 | 2 | GO:0003924 GTPase activity; GO:0007264 small GTPase mediated signal transduction | Hm, Mc, Tc (Ancarola et al., 2017)  Eg (Siles-Lucas et al., 2017)  Fh (Cwikliski et al., 2015) |
| Ras-related protein Rab-14 | W6UPH1 | EUB62681 | 2 | GO:0003924 GTPase activity; GO:0016197 endosomal transport; GO:0031410 cytoplasmic vesicle | Hm, Tc (Ancarola et al., 2017) |
| Uncharacterized protein | W6UNQ7 | EUB63295 | 2 | GO:0030246 carbohydrate binding |  |
| Long-chain-fatty-acid--CoA ligase | W6UTA0 | EUB61592 | 2 | GO:0008152 metabolic process; GO:0016874 ligase activity | Em, Tc (Ancarola et al., 2017) |
| Fructose-bisphosphate aldolase | U6IXV3 | EUB64508 | 2 | GO:0004332 fructose-bisphosphate aldolase activity; GO:0006096 glycolytic process | Em, Hm, Ht, Tc (Ancarola et al., 2017)  Eg (Siles-Lucas et al., 2017)  Fh (Cwikliski et al., 2015)  Ec (Marcilla et al., 2012) |
| Programmed cell death 6-interacting protein (ALIX) | W6VC02 | EUB64384 | 2 |  | Eg (Siles-Lucas et al., 2017) |
| Expressed conserved protein | U6JMR8 | EUB56310 | 2 |  |  |
| Maspardin | W6U7Y4 | EUB56441 | 2 |  |  |
| Vacuolar protein sorting-associated protein | U6J907 | EUB60735 | 2 |  | Em, Mc, Tc (Ancarola et al., 2017) |
| L-lactate dehydrogenase | W6UVI3 | EUB62412 | 2 | GO:0004459 L-lactate dehydrogenase activity; GO:0019752 carboxylic acid metabolic process |  |
| Uncharacterized protein | W6UY44 | EUB63527 | 2 |  |  |
| Alkaline phosphatase | W6U8T0 | EUB56861 | 2 | GO:0004035 alkaline phosphatase activity |  |
| ADP-ribosylation factor 1/3 | W6UFG1 | EUB59848 | 2 | GO:0007264 small GTPase mediated signal transduction | Em, Mc, Tc (Ancarola et al., 2017) |

Ec*, Echinostoma caproni*; Eg, *Echinococcus granulosus*; Em, *Echinococcus multilocularis*; Fh, *Fasciola hepatica*; Hm, *Hymenolepis microstoma*; Ht, *Hydatigera taeniaeformis*; Mc, *Mesocestoides corti*; Tc, *Taenia crassiceps*; Ts, *Taenia solium*.
