# Supplementary material for: Extracellular vesicles from Echinococcus granulosus larval stage: Isolation, characterization and uptake by dendritic cells
Source: PLoS Negl Trop Dis. 2019 Jan 7;13(1):e0007032. doi: 10.1371/journal.pntd.0007032 (PMC6344059; doi:10.1371/journal.pntd.0007032)
Supplement: S2 Table — (DOCX) [file pntd.0007032.s002.docx]

**Supplementary Table 2.** Differencial proteomic analysis of extracellular vesicles from loperamide-treated protoescoleces of *Echinococcus granulosus*.

| **Identified protein** | **Uniprot ID** | **GeneBank ID** | **Peptides** | **Homologous detected in other cestodes** |
| --- | --- | --- | --- | --- |
| Cdc42-interacting protein | W6URG8 | EUB60922 | 12 |  |
| Transforming protein RhoA | W6ULS3 | EUB59107 | 10 | Eg, Mc, Tc (Ancarola et al., 2017)  Eg (Siles-Lucas et al., 2017)  Fh (Cwikliski et al., 2015) |
| Triosephosphate isomerase | U6JN02 | EUB56511 | 10 | Ht, Tc (Ancarola et al., 2017)  Eg (Siles-Lucas et al., 2017)  Ec (Marcilla et al., 2012) |
| 14-3-3 protein epsilon/zeta | U6JEE0 | EUB63343 | 9 | Eg (Siles-Lucas et al., 2017)  Fh (Cwikliski et al., 2015)  Ec (Marcilla et al., 2012) |
| Phospholipid scramblase | W6UV89 | EUB62312 | 8 |  |
| Ferritin | U6JNP4 | EUB56065 | 6 | Ht, Mc, Tc (Ancarola et al., 2017)  Eg (Siles-Lucas et al., 2017)  Fh (Cwikliski et al., 2015) |
| Ras-related protein Rab-7a | W6ULR0 | EUB59087 | 6 |  |
| Serine protease inhibitor | W6V6R7 | EUB62104 | 7 |  |
| Gamma-soluble NSF attachment protein | W6UA62 | EUB58288 | 9 |  |
| Heat shock protein HSP 90-alpha | W6UM50 | EUB54574 | 8 | Eg, Tc (Ancarola et al., 2017)  Eg (Siles-Lucas et al., 2017)  Fh (Cwikliski et al., 2015) |
| Eukaryotic translation initiation factor 5A | W6USI4 | EUB61327 | 6 |  |
| Tubulin beta-3 chain | W6UJD0 | EUB61143 | 7 |  |
| Uncharacterized protein | W6UN14 | EUB62448 | 4 |  |
| Uncharacterized protein | W6U7P9 | EUB56381 | 5 |  |
| Aldehyde dehydrogenase | W6V642 | EUB61869 | 6 |  |
| Dipeptidyl peptidase 3 | U6J0K1 | EUB62208 | 6 |  |
| Non-lysosomal glucosylceramidase | W6UBE4 | EUB58430 | 6 |  |
| Glycerol kinase | W6U277 | EUB55163 | 7 |  |
| Fimbrin | U6IWR5 | EUB64982 | 7 |  |
| Phospholipase D2 | W6UAU2 | EUB58483 | 7 |  |
| Tubulin alpha-1C chain | W6UDI6 | EUB56387 | 7 | Em, Tc (Ancarola et al., 2017)  Eg (Siles-Lucas et al., 2017)  Ec (Marcilla et al., 2012) |
| Alpha-actinin, sarcomeric | W6UY26 | EUB63502 | 7 | Em, Mc, Tc (Ancarola et al., 2017) |
| Glutathione S-transferase | O16058 | AAB66318 | 6 |  |
| Putative UDP-glucose 4-epimerase | W6UN54 | EUB62533 | 6 |  |
| GTPase HRas | W6UDS2 | EUB58966 | 6 | Eg, Tc (Ancarola et al., 2017) |
| Putative phosphoglycerate mutase | W6UW68 | EUB64871 | 6 | Eg (Siles-Lucas et al., 2017)  Ec (Marcilla et al., 2012) |
| Long-chain-fatty-acid--CoA ligase | W6UH87 | EUB60416 | 6 | Em, Tc (Ancarola et al., 2017) |
| Transketolase | U6JBX5 | EUB55174 | 6 | Em, Tc (Ancarola et al., 2017) |
| Ras-related protein RABA1b | W6U1Q9 | EUB54978 | 6 |  |
| Actin-interacting protein | W6USX8 | EUB64418 | 6 |  |
| Rab GDP dissociation inhibitor | U6IW87 | EUB57627 | 6 | Em, Hm, Tc (Ancarola et al., 2017) |
| Tubulin alpha chain | W6UST3 | EUB64685 | 4 |  |
| Asparaginyl-tRNA synthetase, cytoplasmic | W6UMA5 | EUB54629 | 6 |  |
| Ras-related protein Rab-10 | W6UKC0 | EUB61616 | 5 | Eg (Siles-Lucas et al., 2017)  Fh (Cwikliski et al., 2015) |
| Ras protein rab | U6IZC6 | EUB64456 | 4 | Em, Tc (Ancarola et al., 2017) |
| Thioredoxin reductase 3 | W6UPS4 | EUB63258 | 5 |  |
| Acetylcholinesterase | W6UGL0 | EUB57267 | 5 |  |
| Seryl tRNA Synthetase | U6JFG3 | EUB56711 | 5 |  |
| 14-3-3 protein zeta | U6J5Z8 | EUB61917 | 5 | Eg (Siles-Lucas et al., 2017)  Fh (Cwikliski et al., 2015)  Ec (Marcilla et al., 2012) |
| Ras-related protein M-Ras | W6UP09 | EUB62953 | 3 | Hm, Tc (Ancarola et al., 2017) |
| MAGUK p55 subfamily member | W6UZY1 | EUB64127 | 5 |  |
| Serine/threonine-protein kinase N2 | W6V113 | EUB59489 | 5 |  |
| Fatty acid-binding protein | W6UGV8 | EUB60236 | 4 | Mc, Tc, Tp, (Ancarola et al., 2017) |
| Ras protein Rab 2A | U6J6U1 | EUB62853 | 4 | Hm, Tc (Ancarola et al., 2017)  Eg (Siles-Lucas et al., 2017)  Fh (Cwikliski et al., 2015) |
| cGMP-dependent protein kinase, isozyme | W6U8K9 | EUB56756 | 4 | Em, Mc, Tc (Ancarola et al., 2017) |
| Ubiquitin conjugating enzyme E2 N | U6JN29 | EUB59924 | 4 | Em, Tc (Ancarola et al., 2017) |
| Aspartate aminotransferase | W6U899 | EUB57395 | 4 |  |
| Fatty acid binding protein FABP2 | U6JIF2 | EUB56505 | 5 | Mc, Tc, Tp (Ancarola et al., 2017) |
| Coronin | U6J5X0 | EUB61950 | 4 |  |
| Dynein light chain 1, cytoplasmic | W6UHB1 | EUB60443 | 4 | Eg, Mc, Tc (Ancarola et al., 2017) |
| PQ loop repeat containing protein 2 | U6JJC7 | EUB60995 | 5 |  |
| Eukaryotic translation initiation | U6J6D7 | EUB64092 | 4 |  |
| Dihydropyrimidinase | W6UWT9 | EUB62962 | 4 |  |
| Calcium-binding mitochondrial carrier protein SCaMC-1 | W6UAX7 | EUB57691 | 3 |  |
| Charged multivesicular body protein | W6VCG7 | EUB64574 | 4 | Eg (Siles-Lucas et al., 2017)  Fh (Cwikliski et al., 2015) |
| NAD-dependent deacetylase sirtuin-2 | W6UGE3 | EUB60041 | 4 |  |
| Concentrative Na nucleoside cotransporter/ Solute carrier family 28 member 3 | U6JCE6 | EUB60933 | 4 |  |
| Uncharacterized protein | W6U8U0 | EUB56886 | 2 |  |
| Synaptotagmin-2 | W6V0A0 | EUB64282 | 3 | Em, Tc (Ancarola et al., 2017) |
| CRAL-TRIO domain-containing protein C3H8.02 | W6UKV9 | EUB58727 | 4 | Em, Tc (Ancarola et al., 2017) |
| Glutathione S-transferase class-mu isozyme | W6UFZ8 | EUB60405 | 3 |  |
| Aldehyde dehydrogenase =1 | W6USN1 | EUB61382 | 4 |  |
| Superoxide dismutase [Cu-Zn] | W6V988 | EUB63129 | 4 | Tc (Ancarola et al., 2017) |
| ADP-ribosylation factor 6 | W6V0C5 | EUB64312 | 4 | Em, Mc, Tc (Ancarola et al., 2017) |
| Guanine nucleotide-binding protein G(S) subunit alpha | W6UQX7 | EUB60717 | 4 | Em, Tc (Ancarola et al., 2017) |
| Alpha tocopherol transfer protein | U6ITN7 | EUB59143 | 4 |  |
| 1,5-anhydro-D-fructose reductase | W6UTJ3 | EUB56734 | 3 |  |
| Universal stress protein | U6J3T1 | EUB58409 | 3 |  |
| Nascent polypeptide-associated complex subunit alpha, muscle-specific form | W6UUF0 | EUB64301 | 3 | Em, Mc (Ancarola et al., 2017) |
| Tetraspanin | W6UYP7 | EUB63772 | 3 |  |
| Estradiol 17 beta-dehydrogenase | W6U2I8 | EUB55283 | 3 |  |
| Sodium/potassium-transporting ATPase subunit alpha | W6UUP9 | EUB57119 | 4 | Eg, Tc, Ts (Ancarola et al., 2017)  Eg (Siles-Lucas et al., 2017)  Fh (Cwikliski et al., 2015) |
| Epidermal growth factor receptor kinase substrate 8-like protein | W6UFX7 | EUB56987 | 4 | Eg, Hm, Mc (Ancarola et al., 2017) |
| TFIIH basal transcription factor complex helicase subunit | W6VCI4 | EUB64599 | 3 |  |
| Palmitoyltransferase | W6UEY2 | EUB56687 | 4 |  |
| Transforming growth factor-beta-induced protein ig-h3 | W6UFP8 | EUB59736 | 4 |  |
| Coiled-coil and C2 domain-containing protein 1-like protein | W6U675 | EUB56655 | 4 |  |
| Heat shock protein | W6U9X5 | EUB58188 | 4 |  |
| F-actin-capping protein subunit alpha | W6UP73 | EUB62566 | 3 |  |
| Neuronal calcium sensor 2 | W6U4Z2 | EUB56238 | 3 |  |
| Charged multivesicular body protein 4a | U6JBD0 | EUB57410 | 2 |  |
| Scavenger receptor class B member | W6V978 | EUB63119 | 3 |  |
| Sorcin | W6UJK0 | EUB58302 | 2 |  |
| Charged multivesicular body protein | U6JME4 | EUB57191 | 3 | Eg (Siles-Lucas et al., 2017)  Fh (Cwikliski et al., 2015) |
| DnaJ subfamily C member protein | W6UUU7 | EUB64426 | 3 |  |
| Eukaryotic translation initiation factor 2 | U6J7Y0 | EUB62388 | 3 |  |
| Ankyrin repeat containing protein | U6J8Y9 | EUB60751 | 3 | Em, Tc (Ancarola et al., 2017) |
| Proteasome subunit alpha type | U6JH31 | EUB60963 | 3 | Em, Tc (Ancarola et al., 2017)  Eg (Siles-Lucas et al., 2017)  Fh (Cwikliski et al., 2015) |
| Syntaxin-binding protein 5 | W6UR79 | EUB63176 | 3 |  |
| Ras-related protein Rab-27A | W6UCR6 | EUB58526 | 3 | Em, Tc (Ancarola et al., 2017)  Eg (Siles-Lucas et al., 2017)  Fh (Cwikliski et al., 2015) |
| Synaptosomal-associated protein 25-A | W6UDH7 | EUB56377 | 3 |  |
| T-complex protein 1 subunit delta | W6UT85 | EUB64478 | 3 |  |
| Charged multivesicular body protein | U6JCE8 | EUB63889 | 4 | Eg (Siles-Lucas et al., 2017)  Fh (Cwikliski et al., 2015) |
| Prominin-1 | W6UEL6 | EUB56527 | 4 |  |
| Dynein light chain | U6IZK7 | EUB64452 | 2 | Eg, Em, Hm, Mc, Tc (Ancarola et al., 2017) |
| Cyclin-Y-like protein | W6USC8 | EUB64173 | 2 |  |
| DnaJ subfamily A member | W6U753 | EUB57005 | 2 | Eg, Tc (Ancarola et al., 2017) |
| Guanine nucleotide-binding protein subunit beta | W6UI48 | EUB61160 | 3 | Em, Tc (Ancarola et al., 2017) |
| Glucose-6-phosphate isomerase | W6V798 | EUB62329 | 3 |  |
| Uncharacterized protein | W6V2L6 | EUB60184 | 3 |  |
| Eukaryotic translation initiation factor 2 | U6JB55 | EUB58562 | 3 |  |
| Acylphosphatase-2 | W6V9H7 | EUB63279 | 2 |  |
| Actin-depolymerizing factor 2 | W6UJD3 | EUB61251 | 3 | Em, Tc (Ancarola et al., 2017) |
| Glutathione S transferase | U6JFS3 | EUB60467 | 3 |  |
| Endophilin-B1 | W6UDE2 | EUB59340 | 2 | Em, Tc, Ts (Ancarola et al., 2017) |
| Rho GTPase-activating protein | W6U6V4 | EUB54097 | 2 |  |
| Epsin-1 | W6U6X2 | EUB56121 | 3 |  |
| Glucosamine--fructose-6-phosphate aminotransferase [isomerizing] | W6US48 | EUB56254 | 3 |  |
| Rhophilin-2 | W6U351 | EUB55523 | 3 |  |
| Expressed conserved protein | U6IWW9 | EUB63587 | 3 |  |
| Solute carrier family 13 member | W6UCC9 | EUB58910 | 3 |  |
| Charged multivesicular body protein 4a | U6JBD0 | EUB57410 | 2 |  |
| Sodium/hydrogen exchanger | U6J9G0 | EUB56127 | 2 |  |
| Alpha-1,4 glucan phosphorylase/ Glycogen phosphorylase | W6UB03 | EUB58558 | 3 |  |
| Proteasome subunit beta type | U6JD96 | EUB59326 | 2 | Eg (Siles-Lucas et al., 2017)  Fh (Cwikliski et al., 2015) |
| Charged multivesicular body protein 3 | W6V9R5 | EUB63414 | 3 |  |
| Guanylate cyclase | W6UB53 | EUB58325 | 3 |  |
| Uncharacterized protein | W6UCW0 | EUB59135 | 2 |  |
| Actin-related protein 2/3 complex subunit 4 | W6UI15 | EUB61125 | 3 | Hm, Tc (Ancarola et al., 2017) |
| Transitional endoplasmic reticulum ATPase | W6UQD3 | EUB63915 | 3 | Em, Tc (Ancarola et al., 2017)  Eg (Siles-Lucas et al., 2017)  Ec (Marcilla et al., 2012) |
| EH domain-containing protein | U6IWC4 | EUB63467 | 3 |  |
| Golgi-associated plant pathogenesis-related protein | W6U3K0 | EUB55685 | 2 |  |
| Elongation factor 2 | W6UPN1 | EUB63585 | 3 | Em, Mc, Tc (Ancarola et al., 2017) |
| Purine nucleoside phosphorylase/Putative S-methyl-5'-thioadenosine phosphorylase | W6V775 | EUB62299 | 3 |  |
| Zinc transporter | W6UY68 | EUB63547 | 2 |  |
| Expressed conserved protein | U6IYX9 | EUB63589 | 2 |  |
| Serine/threonine-protein phosphatase | W6U419 | EUB55853 | 2 | Hm, Tc (Ancarola et al., 2017) |
| Uncharacterized protein | W6UTA2 | EUB61597 | 2 |  |
| Inner membrane protein yhjX | W6U8I5 | EUB57558 | 2 |  |
| Charged multivesicular body protein | W6U2E6 | EUB54721 | 2 | Em, Tc (Ancarola et al., 2017)  Eg (Siles-Lucas et al., 2017)  Fh (Cwikliski et al., 2015) |
| Uncharacterized protein | W6UT69 | EUB56584 | 2 |  |
| Uncharacterized protein | W6U9F5 | EUB55117 | 2 |  |
| Guanine nucleotide-binding protein G(O) subunit alpha | W6UBY3 | EUB58066 | 2 | Eg (Siles-Lucas et al., 2017)  Fh (Cwikliski et al., 2015) |
| Actin protein 2 B/A | U6J1L9 | EUB64858 | 2 |  |
| Thioredoxin | W6V3D8 | EUB60549 | 2 | Tc, Ts (Ancarola et al., 2017)  Eg (Siles-Lucas et al., 2017)  Fh (Cwikliski et al., 2015) |
| Antigen protein | W6U2H1 | EUB54751 | 2 |  |
| Dynein light chain 1, cytoplasmic | U6JF49 | EUB59668 | 2 | Eg, Tc (Ancarola et al., 2017) |
| Proteasome endopeptidase complex | U6IX51 | EUB58437 | 2 |  |
| Tctex1 domain-containing protein | W6U9J7 | EUB58048 | 2 |  |
| Proteasome endopeptidase complex | W6UJV1 | EUB61333 | 2 |  |
| Inorganic pyrophosphatase | U6J8B6 | EUB64048 | 2 |  |
| Pleckstriny domain-containing family A member | W6VAB9 | EUB63714 | 2 |  |
| Uncharacterized protein | W6UDN8 | EUB55117 | 2 |  |
| Annexin A7 | W6UEP1 | EUB59910 | 2 |  |
| Lysosomal alpha glucosidase | U6JQ59 | EUB56638 | 2 |  |
| Proteasome subunit beta type 6 | U6JCB8 | EUB63335 | 2 |  |
| Guanine nucleotide-binding protein G(Q) subunit alpha | W6UU29 | EUB56894 | 2 |  |
| Programmed cell death protein 6 (PDCD6) | U6JJF9 | EUB60619 | 2 |  |
| Sulfhydryl oxidase | W6UCT4 | EUB59100 | 2 |  |
| Tumor susceptibility gene 101 protein (TSG101) | W6U800 | EUB57290 | 2 |  |
| Eukaryotic translation initiation factor 3 subunit C | W6URL4 | EUB60942 | 2 |  |
| Proteasome subunit beta type-5 | W6U9V0 | EUB57790 | 2 |  |
| E3 ubiquitin protein ligase MARCH8 | U6JAP6 | EUB58278 | 2 |  |
| Protein transport protein Sec61 subunit alpha-like protein | W6V1X1 | EUB64932 | 2 |  |
| Tetraspanin | U6JE59 | EUB54099 | 2 |  |
| Vacuolar protein-sorting-associated protein | W6U562 | EUB56270 | 2 | Em, Mc, Tc (Ancarola et al., 2017) |
| Sorbin | W6UDH4 | EUB56372 | 2 |  |
| Otoferlin | W6V8E9 | EUB62794 | 2 | Em, Tc (Ancarola et al., 2017) |
| Dysferlin | W6UR44 | EUB63693 | 2 |  |
| Dynein light chain 2, cytoplasmic | W6U3G1 | EUB55663 | 2 | Eg, Em, Mc, Tc (Ancarola et al., 2017)  Eg (Siles-Lucas et al., 2017)  Ec (Marcilla et al., 2012) |
| Serine/threonine-protein kinase | W6UKP2 | EUB62105 | 2 |  |
| Expressed conserved protein | U6J7X5 | EUB63367 | 2 |  |
| Uncharacterized protein | W6USF9 | EUB63581 | 2 |  |
| Vacuolar protein sorting-associated protein | W6UMT2 | EUB62368 | 2 | Em, Mc, Tc (Ancarola et al., 2017) |
| FERM domain-containing protein 3 | W6UVH8 | EUB64646 | 2 | Em, Tc (Ancarola et al., 2017) |
| Sn1-specific diacylglycerol lipase beta | W6UXA2 | EUB63167 | 2 |  |
| Serine/threonine-protein phosphatase 2A regulatory subunit A alpha isoform | W6UBZ8 | EUB58918 | 2 |  |
| Vacuolar protein sorting-associated protein 37B | W6UU16 | EUB61872 | 2 |  |
| Expressed conserved protein | U6J321 | EUB62954 | 2 |  |
| Fucosidase alpha L 1 tissue | U6J2X5 | EUB62268 | 2 |  |
| GTP-binding nuclear protein | U6JCJ6 | EUB60932 | 2 | Eg, Tc (Ancarola et al., 2017) |
| Huntingtin-interacting | W6U5E8 | EUB55786 | 2 |  |
| Nardilysin | W6UKZ8 | EUB61748 | 2 |  |
| Nardilysin | W6UTP1 | EUB61747 | 2 |  |
| Exocyst complex component | W6UWQ6 | EUB65091 | 2 |  |
| Ubiquitin hydrolase | W6UII9 | EUB57917 | 2 |  |
| Vacuolar protein-sorting-associated protein 25 | W6U602 | EUB56628 | 2 | Eg (Siles-Lucas et al., 2017)  Fh (Cwikliski et al., 2015) |
| Rab3 | U6JRT3 | EUB61729 | 2 | Eg (Siles-Lucas et al., 2017)  Fh (Cwikliski et al., 2015) |
| Plectin-1 | W6UJ70 | EUB61525 | 2 |  |
| Expressed conserved protein | U6IWR1 | EUB63588 | 2 |  |
| Uncharacterized protein | W6U3J6 | EUB55683 | 2 |  |
| Nucleoporin NUP53 | W6UP25 | EUB60037 | 2 |  |
| Ubiquitin hydrolase | W6UAV1 | EUB58210 | 2 |  |
| Ubiquitin conjugating enzyme E2 L3 | U6J1U6 | EUB64362 | 2 | Em, Tc (Ancarola et al., 2017) |
| 4-hydroxybutyrate coenzyme A transferase | W6UJB6 | EUB61138 | 2 |  |
| E3 ubiquitin-protein ligase CBL-B | W6V060 | EUB64217 | 2 |  |

Ec*, Echinostoma caproni*; Eg, *Echinococcus granulosus*; Em, *Echinococcus multilocularis*; Fh, *Fasciola hepatica*; Hm, *Hymenolepis microstoma*; Ht, *Hydatigera taeniaeformis*; Mc, *Mesocestoides corti*; Tc, *Taenia crassiceps*; Ts, *Taenia solium*; Tp, *Taenia pisiformis.*
