# Supplementary material for: Extracellular vesicles from Echinococcus granulosus larval stage: Isolation, characterization and uptake by dendritic cells
Source: PLoS Negl Trop Dis. 2019 Jan 7;13(1):e0007032. doi: 10.1371/journal.pntd.0007032 (PMC6344059; doi:10.1371/journal.pntd.0007032)
Supplement: S3 Table — (DOCX) [file pntd.0007032.s003.docx]

| **Uniprot ID** | **Identified Domains (CDART, ProDom, CDD)** | **Domain position** | **Protein length (aa)** | **Sec-P**  **Score**  **(>0.6)/ Signal P** |
| --- | --- | --- | --- | --- |
| W6U780 | RecF/RecN/SMC (structural maintenance of chromosomes) N terminal domain (cl25732) | 758-899 | 1019 | *0.745/- |
| W6UFB0 | Spore_III_AB Superfamily  (cl08022) | 1108-1278 | 1685 | 0.466/- |
| W6ULE5 | Antigenic protein of *Taenia solium* (TSES33, GP50C and TSES38; PDA1F0X2) | 1-309 | 595 | 0.299/- |
| W6U6P0 | Antigenic protein of *Taenia solium* (TSES33, GP50C and TSES38; PDA1F0X2) | 1-309 | 344 | 0.585/+ |
| W6UE44 | No putative conserved domains |  | 388 | 0.303/- |
| W6VBX7 | Dynein light chain superfamily (cl03131)  Calcium-tegumental antigen (PD006693) | 80-164; 325-409  355-410 | 415 | 0.563/- |
| W6UY44 | No putative conserved domains |  | 465 | 0.505/- |
| W6UX03 | Uncharacterized protein of *Schistosoma mansoni* (PDA8Z8Y0; PDD2K269)  Uncharacterized protein of *Clonorchis sinensis* (PDC8Y4T8)  Bravo_FIGEY Superfamily (cl16451) | 605-1206  1308-1429  1366-1439 | 1482 | 0.182/- |
| W6U1N0 | Tetraspanin LEL Superfamily (cl02781) | 51-248 | 293 | 0.924/+ |
| W6UNQ7 | GLECT Superfamily (Galectin/galactose-binding lectin, cl00071) | 16-143 | 1291 | 0.097/- |
| W6UJ45 | Antigenic protein of *Taenia solium* (TSES33, GP50C and TSES38; PDA1F0X2) | 1-129 | 187 | 0.717/+ |
| W6U6B2 | Antigenic protein of *Taenia solium* (TSES33, GP50C and TSES38; PDA1F0X2) | 18-121 | 121 | 0.740/+ |
| W6UFB6 | No putative conserved domains |  | 258 | 0.377/- |
| W6U7G9 | No putative conserved domains |  | 234 | 0.380/- |
| W6UN14 | Uncharacterized protein of *Taenia solium* (PD005232) | 32-212 | 269 | * 0.906 |
| W6U7P9 | No putative conserved domains |  | 124 | 0.753/+ |
| W6UCY9 | HAGH-C Superfamily (Hydroxyacylglutathione hydrolase C-terminus, cl25711) | 10-244 | 245 | 0.379/- |
| W6U828 | Thioredoxin like Superfamily (cl0388) | 2-97 | 108 | *0.774/- |
| W6U8U0 | Antigenic protein of *Taenia solium* (TSES33, GP50C and TSES38; PDA1F0X2) | 1-232 | 316 | 0.776/+ |
| W6TZB6 | Antigenic protein of *Taenia solium* (TSES33, GP50C and TSES38; PDA1F0X2) | 3-200 | 200 | *0.676/- |
| W6UIJ6 | Uncharacterized protein of *Schistosoma mansoni* (PDC7V7P6)  Transferase acid metal-binding zinc-finger zinc farnesoic of *Taenia solium* (PD293778) | 1-78  95-152 | 155 | 0.324/- |
| W6V2L6 | No putative conserved domains |  | 303 | *0.726/- |
| W6UT69 | MIP-T3 Superfamily ( Microtubule-binding protein MIP-T3, cl25761) | 103-292 | 493 | 0.101/- |
| W6UCW0 | No putative conserved domains |  | 423 | 0.547/- |
| W6UDN8 | No putative conserved domains |  | 717 | 0.273/- |
| W6U9F5 | RecF/RecN/SMC (structural maintenance of chromosomes) N terminal domain (cl25732)of | 217-271 | 374 | 0.380/- |
| W6UTA2 | Tetraspanin LEL Superfamily (cl02781) | 1-77 | 78 | 0.181/- |
| W6USF9 | Thioredoxin like Superfamily (cl00388) | 17-163 | 198 | *0.616/- |
| W6U3J6 | No putative conserved domains |  | 299 | 0.375/- |
| U6JMR8 | No putative conserved domains |  | 207 | 0.374/- |
| U6IWW9 | No putative conserved domains |  | 321 | 0.316/- |
| U6IYX9 | No putative conserved domains |  | 647 | 0.386/- |
| U6J7X5 | No putative conserved domains |  | 241 | *0.643/- |
| U6J321 | No putative conserved domains |  | 422 | 0.432/- |
| U6IWR1 | No putative conserved domains |  | 444 | *0.754/- |

**Supplementary Table 3.** Uncharacterized protein and expressed conserved protein identified from *Echinococcus granulosus* extracellular vesicles.

SecretomeP: Secretion prediction according to SecretomeP 2.0 server. Protein sequences were analyzed in terms of mammalian secretion pathways (>0.6). Numbers correspond to NN-score.

SignalP: Secretion prediction according to Signal 4.0 server. +/- indicates presence/absence of signal peptide, respectively.

* indicates protein secretion by non-classical pathway
