# Supplementary figures and images for: Extracellular vesicles from Echinococcus granulosus larval stage: Isolation, characterization and uptake by dendritic cells
Source: PLoS Negl Trop Dis. 2019 Jan 7;13(1):e0007032. doi: 10.1371/journal.pntd.0007032 (PMC6344059; doi:10.1371/journal.pntd.0007032)

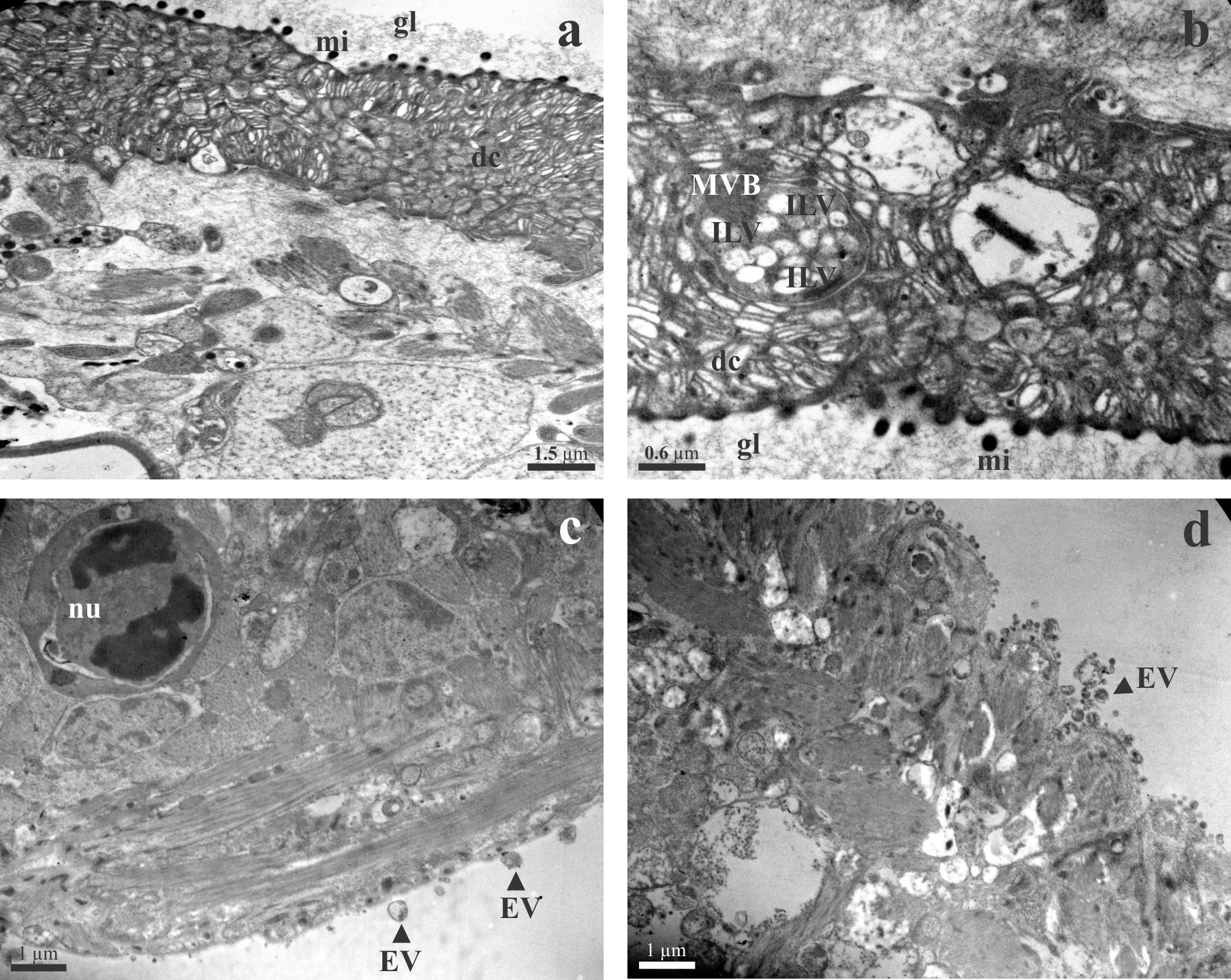

Supplement: S1 Fig — (a-b) Control (c-d) loperamide-treated protoscoleces (20 μM, 18 h). MVB, multivesicular bodies; ILV, intraluminal vesicles; EV and arrowhead, extracellular vesicle; n, nucleus; dc, distal cytoplasm; gl, glycocalyx; mi, microtriches. (TIF) [file pntd.0007032.s006.tif]

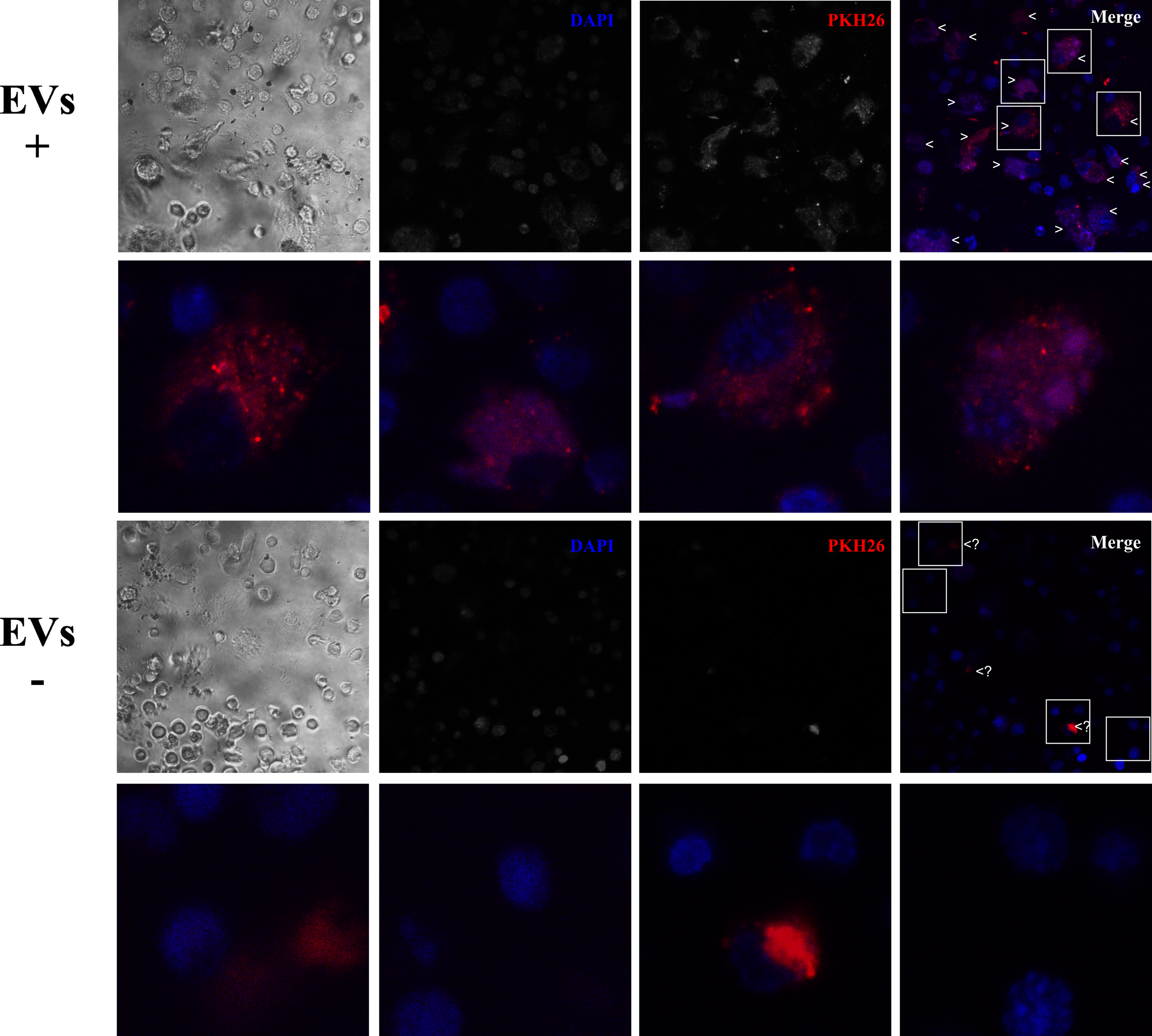

Supplement: S2 Fig — 1x10^6^ cells/ml Bone Marrow Dendritic Cells (BMDCs) obtained after 6 days of culture in presence of FLT3-L were incubated with EVs labeled with PKH26 (EVs +) or with PKH26 dye alone purified without EVs (EVs -). Almost 40% of cells incubated with labeled EVs show positive staining (homogeneous fluorescent pattern with punctate dot structures, indicated by arrowheads), while only 3% of them were stained with a diffuse pattern in presence of the dye alone (indicated by an arrowhead and a question mark). Boxed areas correspond to the amplified images. (TIF) [file pntd.0007032.s007.tif]

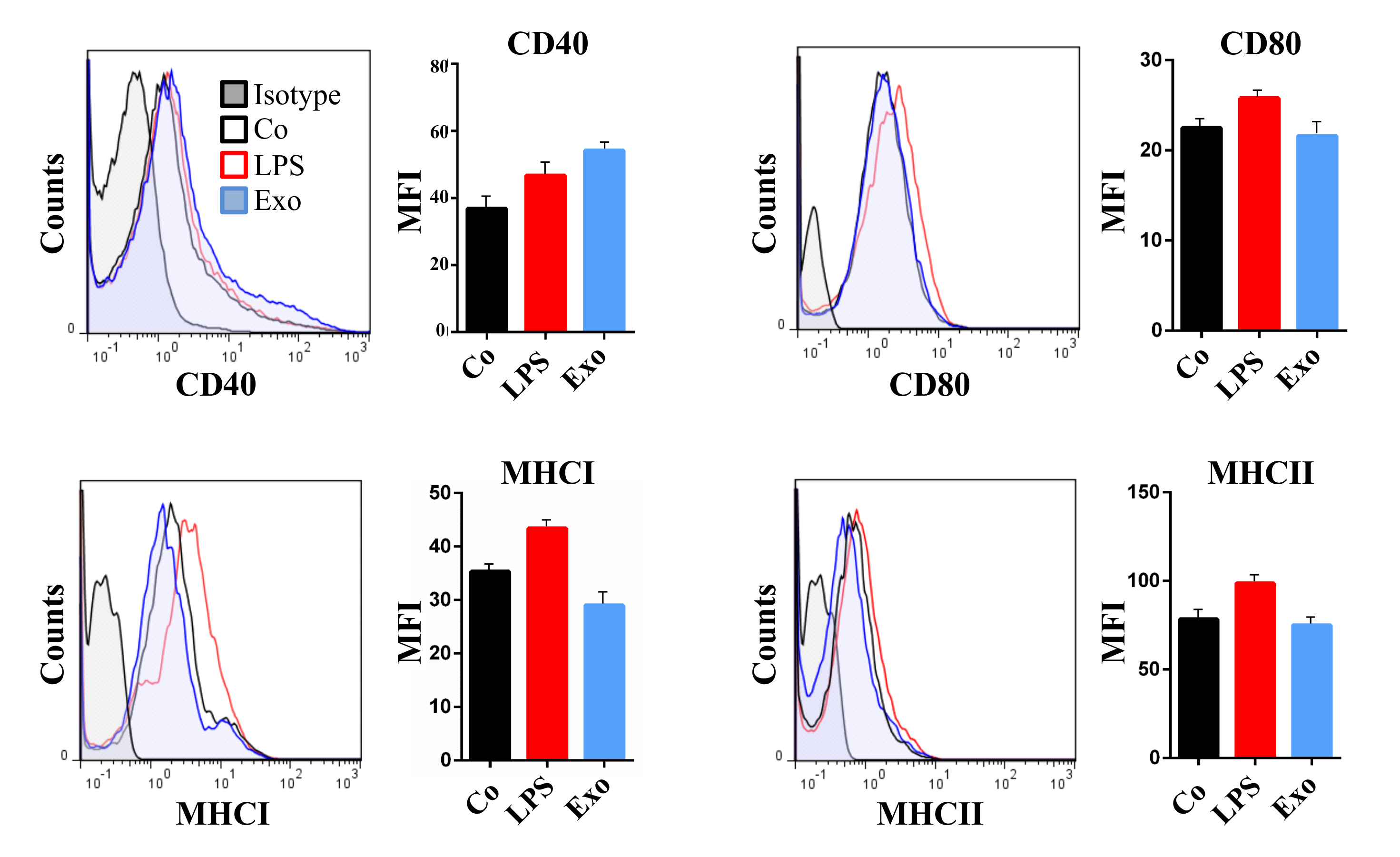

Supplement: S3 Fig — 1x10^6^ cells/ml Bone Marrow Dendritic Cells (BMDCs) were obtained after 6 days of culture in presence of FLT3-L. After 18 h, BMDCs maturation was analyzed by flow cytometry by studying the difference in mean fluorescent intensity (MFI) of CD40, CD80, MHCI and MHCII in the gate of CD11c+ cells by histograms. A representative experiment (n = 3) is shown. Data were presented as the mean ± SD. No significant differences were detected using a Kruskal-Wallis with Dunn's post-test, * p < 0.05. (TIF) [file pntd.0007032.s008.tif]
